# Targeted delivery of fat extract by platelet membrane-cloaked nanocarriers for the treatment of ischemic stroke

**DOI:** 10.1186/s12951-022-01461-2

**Published:** 2022-05-31

**Authors:** Cheng Wang, Xuewei Yang, Yixu Jiang, Lin Qi, Deli Zhuge, Tongtong Xu, Yiyan Guo, Mingwu Deng, Wenjie Zhang, Dongyan Tian, Qingqing Yin, Li Li, Zhijun Zhang, Yongting Wang, Guo-Yuan Yang, Yijie Chen, Yaohui Tang

**Affiliations:** 1 School of Biomedical Engineering, Med-X Research Institute, Shanghai Jiao Tong University, 1954 Hua Shan Road, Shanghai, 200030 China; 2 Department of Obstetrics and Gynecology, the Second Affiliated Hospital of Wenzhou Medical University, 109 Xueyuan West Road, Wenzhou, 325027 China; 3 Department of Plastic and Reconstructive Surgery, Shanghai Key Laboratory of Tissue Engineering, Shanghai 9th People’s Hospital, Shanghai Jiao Tong University, 639 Zhi Zao Ju Road, Shanghai, 200011 China

**Keywords:** Angiogenesis, Fat extract, Ischemic stroke, Platelet membrane, Neurogenesis

## Abstract

**Background:**

Our previous studies suggest that human fat extract (FE) contains a variety of angiogenic factors and may provide an alternative treatment option for stroke. However, the therapeutic effect is largely limited due to its short half-life, and inaccurate targeting.

**Results:**

Herein, we leverage the targeting abilities of platelets (PLTs) to the lesion area of stroke and Arg-Gly-Asp (RGD) peptides to the angiogenic blood vessels to develop a biomimetic nanocarrier that capable of delivering FE precisely to treat stroke. The biomimetic nanocarriers are comprised of FE-encapsulated PLGA (poly(lactic-co-glycolic acid)) core enclosed by RGD peptides decorated plasma membrane of PLTs, namely RGD-PLT@PLGA-FE. We found that RGD-PLT@PLGA-FE not only targeted damaged and inflamed blood vessels but also achieved rapid accumulation in the lesion area of ischemic brain. In addition, RGD-PLT@PLGA-FE kept a sustained release behavior of FE at the lesion site, effectively increased its half-life and promoted angiogenesis and neurogenesis with delivering neurotrophic factors including BDNF, GDNF and bFGF to the brain, that ultimately resulted in blood flow increase and neurobehavioral recovery.

**Conclusions:**

In conclusion, our study provides a new strategy to design a biomimetic system for FE delivery and it is a promising modality for stroke therapy.

**Graphical Abstract:**

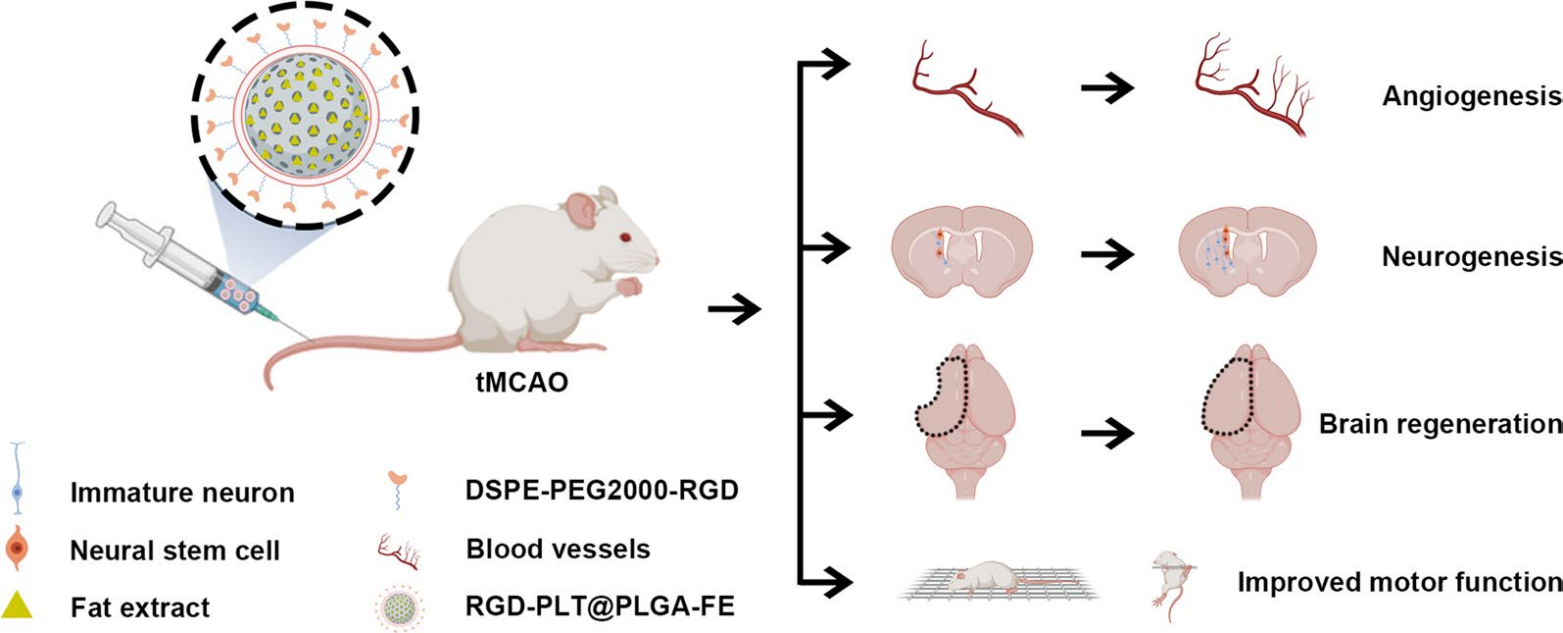

**Supplementary Information:**

The online version contains supplementary material available at 10.1186/s12951-022-01461-2.

## Background

Stroke is the second leading cause of disability and mortality in the world, with rare therapeutic options[1–3]. Tissue plasminogen activator(t-PA) is currently the only thrombolytic drug approved by the FDA. However, the use of t-PA is hindered by its limited therapeutic time window (4.5 h) and the risk of hemorrhage, thus only less than 5% of stroke patients can benefit from this therapy[4]. Therefore, developing new strategies for treating stroke are in great need.

Stem cell-based therapy represents a potential therapeutic strategy for stroke[5, 6]. Among the stem cell types that have been explored in animal studies and clinical trials, adipose derived stem cells (ADSCs) have attracted great attention due to their ease of isolation, abundance, and differentiation capacity. Amounting evidence shows the therapeutic effects of ADSCs in various diseases[7, 8]. However, ADSCs derived from different donors show high heterogeneity [9], which could affect the features such as proliferative capacity, differentiation potential and immunophenotype etc. [9, 10]. For this reason, the clinical application of ADSCs has been greatly restricted[11], and more efficient solutions are needed. Previous studies highlighted that the therapeutic effects of ADSCs are primarily through secretion of growth factors rather than in-situ differentiation to replace dead cells. Thus, administration of ADSCs-derived factors has recently been considered as an alternative strategy, which can overcome many safety concerns and limitations of stem cell-based therapy[12–16]. However, the process of collecting ADSCs-derived factors is complicated and labor-intensive, and the quality varies greatly between different batches. To overcome these issues, we have recently reported the extraction of cell-free liquid fraction, termed fat extract (FE), from human fat using a mechanical approach[17, 18]. FE is a cell-free fraction that avoids cell-related safety concerns and has better clinical applicability. Compared to stem cells, FE is easier to collect and can be stored for a longer time, and has lower immunogenicity[18]. In addition, FE contains a variety of angiogenic factors, including VEGF, TGF-β, bFGF, and GDNF etc., and injection of FE could enhance fat graft survival via proangiogenic, anti-apoptotic and pro-proliferative effects in a mouse fat graft model, as well as increase angiogenesis in hindlimb ischemic model[17]. However, it also has several limitations that impede its clinical translation. First, the bioavailability of FE is short. Thus, it needs to be injected frequently to achieve long-term therapeutic effect. Second, FE is usually injected in situ as it has no targeting ability. Recently, natural cell membrane-coated nanoparticles have been developed as drug carriers to preferentially target disease sites. For example, paclitaxel (PTX)-encapsulated polycaprolactone (PCL) nanoparticles camouflaged by red blood cell membrane with CRGD[K/R]GP[D/E]C (iRGD) modification showed prolonged blood circulation that have been utilized for metastatic breast cancer treatment[19]. Nanoparticles loaded with indocyanine green (ICG) and further coated with cancer cell membrane showed specific homologous targeting ability to cancer cells, resulting in a preferable photothermal response and favored to fluorescence/photoacoustic (FL/PA) imaging [20]. It not only allows real-time monitoring, but also has the therapeutic effect on slowing down cancer growth. Neutrophil membrane-derived nanovesicles loaded with Resolvin D2 (RvD2) can target to the inflammation areas and enhance retention, thus protecting brain damage during ischemic stroke[21]. Among these membrane-coated particles, platelets (PLTs) membrane coated nanoparticles showed unique targeting abilities. First, PLTs have a circulation half-life of approximately 30 h, which is attributable to the CD47 for inhibiting immune clearance. Second, PLTs also express a variety of receptors such as integrins and selectins that dynamically adhere to damaged vasculature[22]. With such broad and dynamic biointerfacing capabilities, PLTs membrane coated nanoparticles have become attractive drug carriers for targeted delivery to inflamed blood vessels[23, 24].

Here, we designed a PLTs membrane-cloaked PLGA nanoparticle loaded with FE and conjugated with RGD peptide (namely RGD-PLT@PLGA-FE) for targeted delivery of FE into the infarct area of stroke mice. RGD-PLT@PLGA nanoparticles were used to deliver FE to promote angiogenesis and neurogenesis to repair the damaged brain. As a consequence of the mimicking properties of PLTs membrane and RGD peptides, the nanoparticles enhanced the circulation time and behaved actively targeting capability to the ischemic stroke areas. What’s more, due to the degradability of PLGA, FE could be sustainly released to treat stroke. When the bioengineered, targeted, sustained release nanoparticles were actively delivered to the ischemic area, angiogenesis and neurogenesis were enhanced, thus achieving effective therapeutic efficacy for ischemic brain.

## Results

### Preparation and characterization of RGD-PLT@PLGA-FE.

**Fig. 1:**
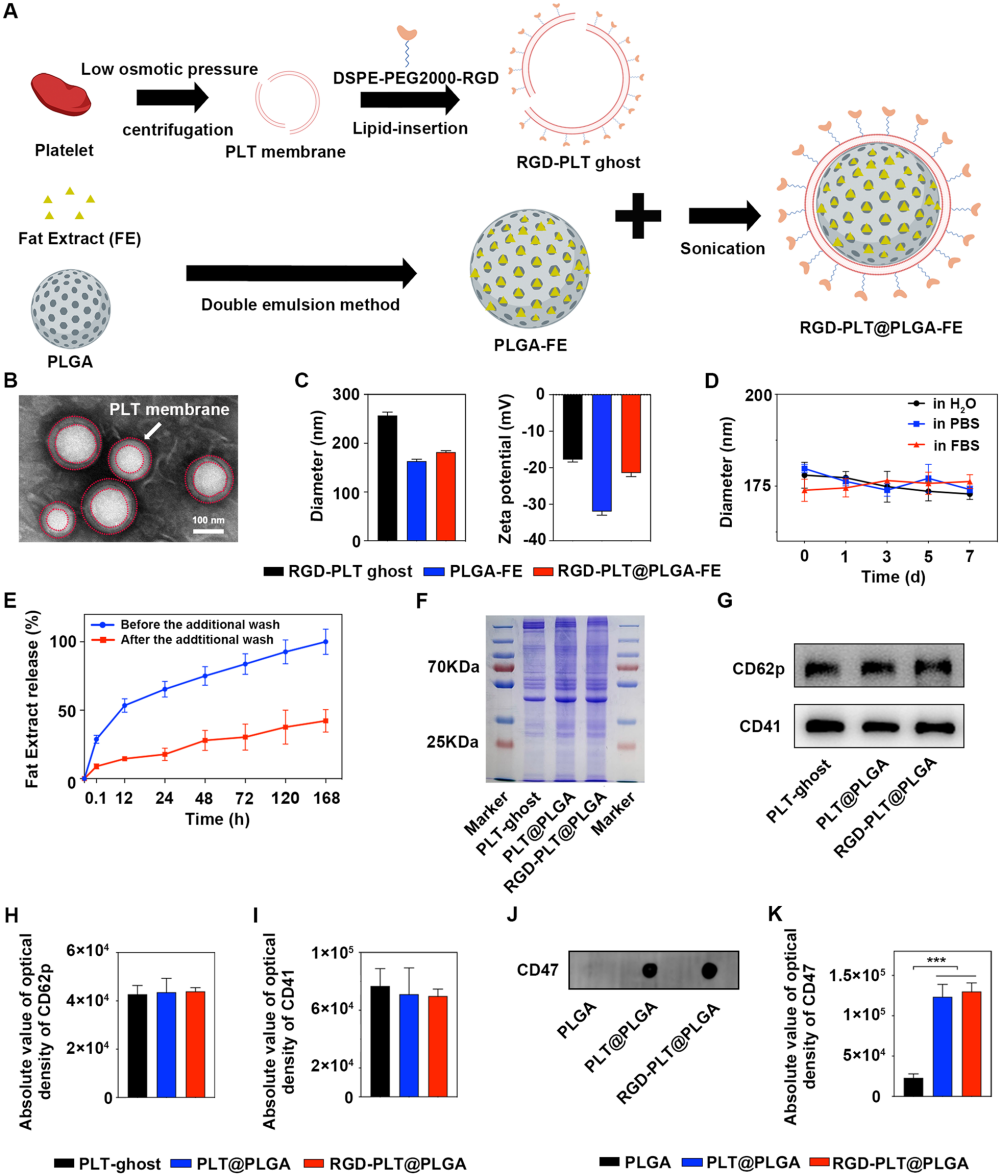
Characterization of RGD-PLT@PLGA-FE. **A** Schematic diagram of the preparation of RGD-PLT@PLGA-FE. **B** Representative TEM image of RGD-PLT@PLGA-FE. Scale Bar = 100 nm. Red circles showed PLT membranes that coated at the surface of PLGA nanoparticles. **C** The hydrodynamic diameters and Zeta potentials of the PLT membrane vesicles, PLGA-FE and RGD-PLT@PLGA-FE. n = 3/group. **D** The stability of RGD-PLT@PLGA-FE in water, PBS and 50% FBS. n = 3/group. **E** Release curve of FE from RGD-PLT@PLGA-FE before and after the additional wash. n = 3/group. **F** Protein composition of platelet vesicle (PLT-ghost), PLT@PLGA, and RGD-PLT@PLGA analyzed by Coomassie staining. **G.** CD62p and CD41 expression in PLT-ghost, PLT@PLGA, and RGD-PLT@PLGA determined by Western blot. n = 3/group. **H** Quantitative analysis of expression of CD62p in PLT-ghost, PLT@PLGA and RGD-PLT@PLGA. n = 3/group. **I** Quantitative analysis of expression of CD41 in PLT-ghost, PLT@PLGA and RGD-PLT@PLGA. n = 3/group. **J** Dot blot analysis of extracellular immunoglobulin domain of CD47 in PLGA only, PLT@PLGA, RGD-PLT@PLGA. **K** Quantitative analysis of extracellular immunoglobulin domain of CD47 in PLGA, PLT@PLGA and RGD-PLT@PLGA. n = 3/group. Data presented as mean ± SD. ***p < 0.001

FE was harvested according to our previous study and encapsulated into biodegradable PLGA nanoparticle cores via a double emulsion process (Fig. 1A). The optimized FE loading efficiency (~ 58%) and capacity (~ 4.3%) were determined when the initial input of FE was up to 30 mg/ml (total protein concentration) (Additional file 1: Fig S1). Meanwhile, to fabricate the functionalized membrane, the maleimide group of DSPE-PEG2000-Mal was first reacted with the thiol group of RGD at a molar ratio of 1: 1 to form DSPE-PEG2000-RGD, which was incorporated onto the mouse PLTs membrane at 5% (w/w) ratio via lipid-insertion method. RGD-PLT@PLGA-FE was then prepared by sonicating RGD modified PLTs membrane with FE-loaded PLGA cores, and their successful coating was analyzed through physicochemical characterizations. As shown in Fig. 1B, transmission electron microscopy (TEM) images presented the surface morphology of RGD-PLT@PLGA-FE with a typical shell-core structure, in which PLGA core with bright color was wrapped by a layer of the membrane (red dashed circles). Compared with PLGA-FE (163.33 ± 4.04 nm), the hydrodynamic diameter of RGD-PLT@PLGA-FE was increased to 181.86 ± 2.97 nm measured by dynamic light scattering (DLS) (Fig. 1C), indicating the successful coating, in agreement with previous studies[25, 26]. In addition, Zeta potential of PLGA with a surface charge of -32 mV was increased to − 17 mV after PLTs membrane coating, which was comparable to that of PLTs vesicles (Fig. 1C), suggesting PLTs membrane has cloaked onto the surface of PLGA core successfully. Over time, the hydrodynamic diameters of RGD-PLT@PLGA-FE nearly kept consistent for 7 days both in water, 1 × PBS and 50% fetal bovine serum solution (FBS) (Fig. 1D). While bare PLGA aggregated quickly up to micro-scale when transferred into 50% FBS due to the electrostatic shielding effect and the diameter kept increase over 3 days (Additional file 1: Fig S7), which demonstrated the stability of RGD-PLT@PLGA-FE. In parallel, FE can be released as time elapsed at planned time points including 0 h, 0.1 h, 12 h, 24 h, 48 h, 72 h, 120 h, and 168 h before and after the additional wash. We found that a burst release of FE before the additional wash, which may be attritubed to the adsorption of FE on the surface or insertion in the superficial of PLGA. However, after three additional washes, a controlled release of protein from RGD-PLT@PLGA-FE, rather than a burst release manner was observed (Fig. 1E). Coomassie brilliant blue staining showed protein profiles released from PLGA-FE exhibited similar to free FE at an equivalent protein concentration (Additional file 1: Fig S6A), indicating synthesis of PLGA-FE did not affect the protein compositions of FE. ELISA assay revealed that GDNF, bFGF, TGF-β and VEGF in PLGA-FE displayed comparable levels to those in free FE (Additional file 1: Fig S6B). Importantly, coomassie brilliant blue staining and Western Blot results showed PLTs membrane-related surface proteins including CD62p and CD41 were still displayed in RGD-PLT@PLGA-FE nanoparticles (Fig. 1F and G) and no significantly difference among the three groups (Fig. 1H and I), warranting further investigation for their targeting and therapeutic role in stroke. High-performance liquid chromatography (HPLC) showed that the conjugation rate of DSPE-PEG2000-MAL and RGDyC was 20.69% ± 2.27% (Additional file 1: Fig S3A and B). Microplate reader indicated that the percentage of conjugated (FITC) RGDyC onto nanoparticles was 11.93% ± 3.09% (Additional file 1: Fig S3D), proving the insertion of DSPE-PEG2000-RGD into the PLT@PLGA. Dot Blot assay demonstrated that the signal was aggregated on both PLT@PLGA and RGD-PLT@PLGA (Fig. 1J and K), which demonstratted the correct orientation of the integrated PLT membrane proteins.

### In Vitro effect of RGD-PLT@PLGA-FE on tube formation and migration of HUVECs

**Fig. 2:**
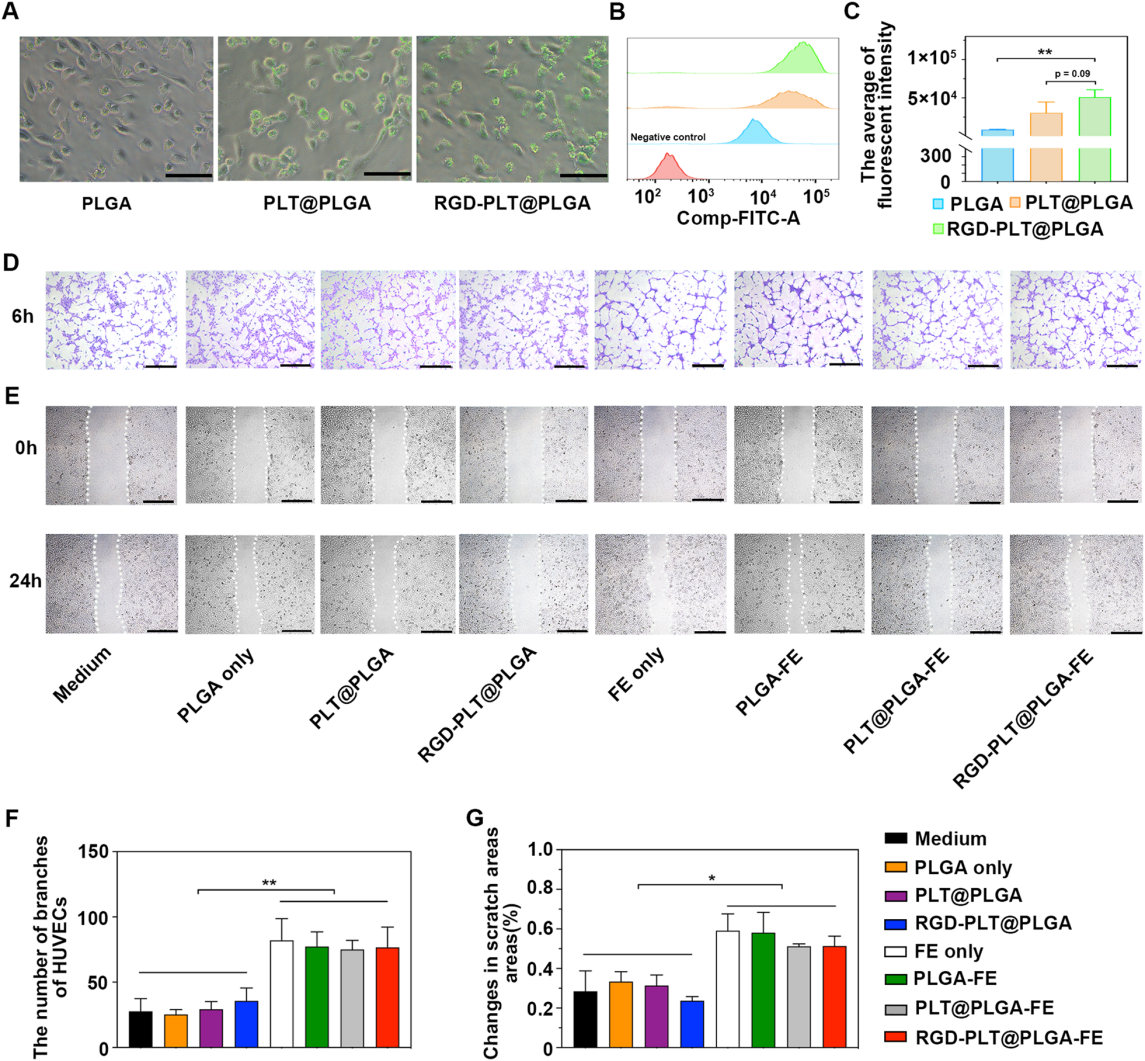
Effects of RGD-PLT@PLGA-FE on HUVECs in Vi*tro*. **A** The overlap imaging of HUVECs treated with DiO labeled PLGA, PLT@PLGA and RGD-PLT@PLGA (green). Scale Bar = 100 μm. **B**, **C** Flow cytometry showed the average fluorescent intensity of HUVECs incubated with DiO labeled PLGA, PLT@PLGA and RGD-PLT@PLGA. n = 3/group. **D** Tube formation of HUVECs that treated with medium, FE, PLGA, PLGA-FE, PLT@PLGA, PLT@PLGA-FE, RGD-PLT@PLGA, RGD-PLT@PLGA-FE. Scale Bar = 500 μm. **E** Migration of HUVECs that treated with medium, FE, PLGA, PLGA-FE, PLT@PLGA, PLT@PLGA-FE, RGD-PLT@PLGA, RGD-PLT@PLGA-FE. Scale Bar = 500 μm. **F** Quantitative analysis of the number of the branches of HUVECs that treated with medium, FE, PLGA, PLGA-FE, PLT@PLGA, PLT@PLGA-FE, RGD-PLT@PLGA, RGD-PLT@PLGA-FE. n = 3/group. **G.** Quantitative analysis of the changes in scratch areas that treated with medium, FE, PLGA, PLGA-FE, PLT@PLGA, PLT@PLGA-FE, RGD-PLT@PLGA, RGD-PLT@PLGA-FE. n = 3/group. Data presented as mean ± SD. *p < 0.05, **p < 0.01

To evaluate the targeting ability of RGD-PLT@PLGA-FE toward inflamed endothelial cells and their effects on tube formation and migration of HUVECs in vitro, HUVECs were treated with LPS (2 µg/ml) to mimic in *vivo* inflammation and then incubated with 3,3′-dioctadecyloxa carbocyanine perchlorate (DiO) labeled-PLGA, PLT@PLGA and RGD-PLT@PLGA, respectively. The fluorescence imaging and flow cytometry results showed that much more PLT@PLGA particles were uptake by HUVECs, compared to PLGA particles, suggesting PLTs coating increased the targeting ability of PLGA particles to inflamed endothelial cells (Fig. 2A, B and C). In addition, compared to PLT@PLGA, more RGD-PLT@PLGA particles were uptake by HUVECs, indicating RGD conjugation can further increase their targeting ability.

Tube formation and migration assay were performed to examine the angiogenic effect of RGD-PLT@PLGA-FE on HUVECs. Tube formation assay revealed that FE only, PLGA-FE, PLT@PLGA-FE, and RGD-PLT@PLGA-FE treatments all increased the number of branches and tube-like structures in HUVECs compared with that of FE-free controls (Fig. 2D and F). Cell migration assay showed that FE, PLGA-FE, PLT@PLGA-FE, and RGD-PLT@PLGA-FE treatment increased the migration of HUVECs, in comparison to that of controls (Fig. 2E and G). As shown in Additional file 1: Fig S5**,** FE released from PLGA-FE, PLT@PLGA-FE, or RGD-PLT@PLGA-FE increased HUVECs viability in 24 h, 48 h, 72 h and 96 h. These results indicated that FE released from nanoparticles is able to enhance the tube formation and migration of HUVECs, with the similar effect as free FE.

### In vivo fluorescence imaging of injected RGD-PLT@PLGA in ischemic stroke mice

**Fig. 3:**
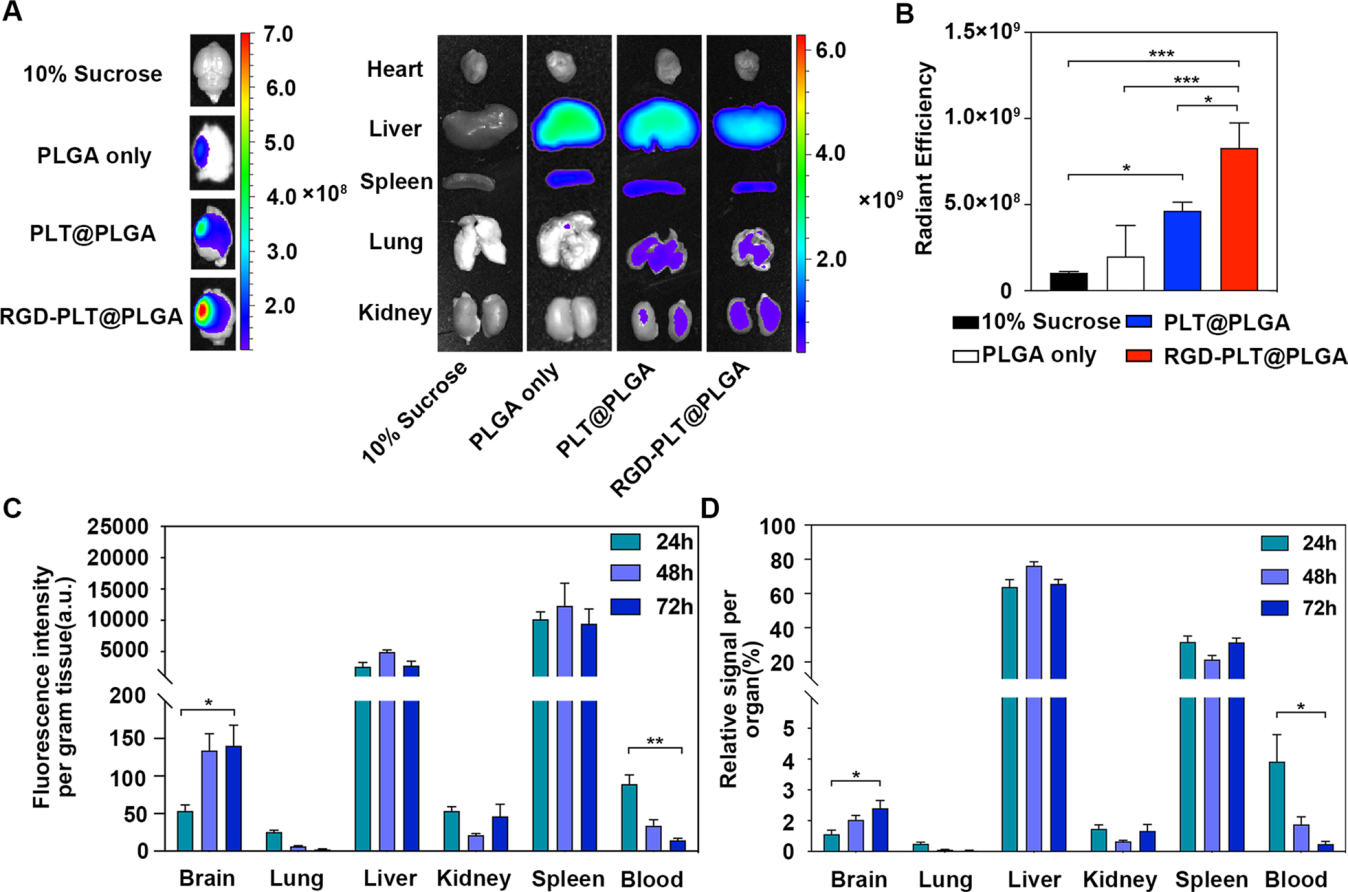
In *vivo* fluorescence imaging of stroke mice treated with RGD-PLT@PLGA. **A** EX vivo fluorescence imaging of heart, liver, spleen, lung and kidney of mice that treated with 10% sucrose, PLGA only, PLT@PLGA and RGD-PLT@PLGA. **B** Quantitative analysis of the average DiD fluorescence signal intensities in the brain that treated with 10% sucrose, PLGA, PLT@PLGA and RGD-PLT@PLGA. n = 3/group. **C** Quantitative analysis of the average DiD fluorescence signal intensity per gram tissue after injection with RGD-PLT@PLGA over time (24 h, 48 h and 72 h). n = 3/group. **D.** Quantitative analysis of the relative signal per organ after injection with RGD-PLT@PLGA over time (24 h, 48 h and 72 h). n = 3/group. Data presented as mean ± SD. *p < 0.05, **p < 0.01, ***p < 0.001

In order to investigate the biodistribution of RGD-PLT@PLGA, 1,1’-Dioctadecyl-3,3,3′,3′-Tetramethylindodicarbocyanine (DiD) labeled PLGA particles were injected into ischemic mice by tail vein. Fluorescence imaging was used to capture the fluorescence images of DiD-labeled PLGA particles in organs including brain, heart, liver, spleen, lung and kidney of mice at 24 h after injection. As shown in Fig. 3A, the fluorescent intensity of PLT@PLGA in the ischemic lesion of the brain was higher than that of non-coated PLGA, suggesting that PLT coating increased the targeting ability of PLGA to the brain. More importantly, conjugation of RGD to PLT@PLGA further increased its targeting to lesion area of stroke brain, and decreased its accumulation in other organs (Fig. 3B. and Additional file 1: Fig S4). We further evaluated the distribution of DiD-labeled RGD-PLT@PLGA in different organs over time (24 h, 48 h and 72 h) by measuring the fluorescent intensities in different organs. The results showed that fluorescent intensity in brain was significantly increased from 24 to 72 h after injection (Fig. 3C and D), and the fluorescent intensity of blood significantly reduced at 72 h compared with that at 24 h. Besides, a considerable amounts of RGD-PLT@PLGA were arrested in the reticuloendothelial system (RES) including liver and spleen, in agreement with other published studies[27].

### RGD-PLT@PLGA-FE treatment improved behavioral recovery and reduced brain atrophy volume

**Fig. 4:**
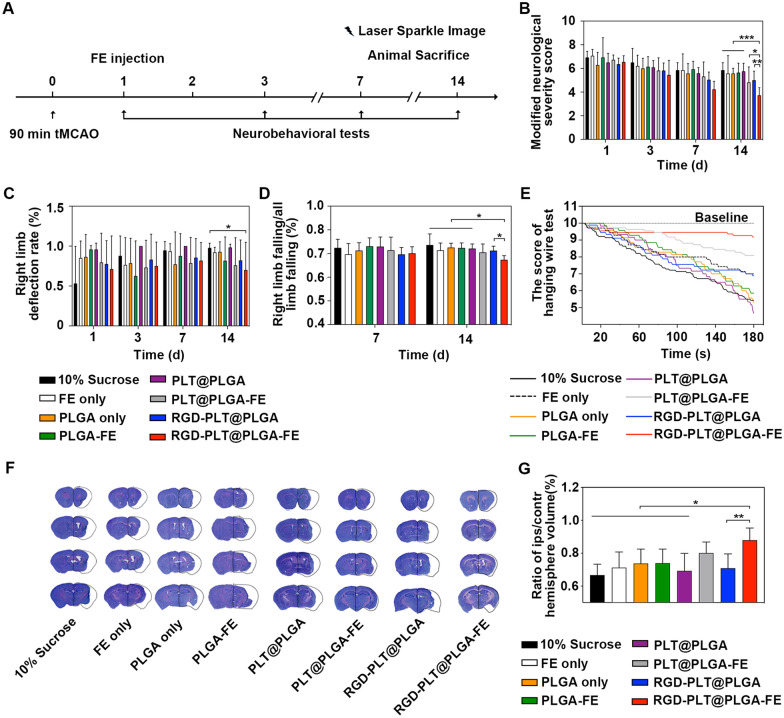
Evaluation of neurobehavioral recovery and brain atrophy volume of ischemic stroke mice. **A** The experimental scheme. **B** Modified neurological severity score (mNSS) of mice that treated with 10% sucrose, FE, PLGA, PLGA-FE, PLT@PLGA, PLT@PLGA-FE, RGD-PLT@PLGA and RGD-PLT@PLGA-FE. n = 14, 7, 7, 7, 6, 11, 11, 11 (from left to right). **C** Elevated body swing test of mice that treated with 10% sucrose, FE, PLGA, PLGA-FE, PLT@PLGA, PLT@PLGA-FE, RGD-PLT@PLGA, RGD-PLT@PLGA-FE. n = 14, 7, 7, 6, 6, 11, 10, 11 (from left to right). **D** Grid walking test of mice that treated with 10% sucrose, FE, PLGA, PLGA-FE, PLT@PLGA, PLT@PLGA-FE, RGD-PLT@PLGA, RGD-PLT@PLGA-FE. n = 10, 7, 7, 7, 6, 9, 8, 10 (from left to right). **E** Hanging wire test of mice that treated with 10% sucrose, FE, PLGA, PLGA-FE, PLT@PLGA, PLT@PLGA-FE, RGD-PLT@PLGA, RGD-PLT@PLGA-FE. n = 14, 7, 7, 7, 6, 11, 9, 11 (from left to right). **F** Representative cresyl violet-stained brain sections that treated with 10% sucrose, FE, PLGA, PLGA-FE, PLT@PLGA, PLT@PLGA-FE, RGD-PLT@PLGA, RGD-PLT@PLGA-FE at 14 days after stroke. **G** Quantitative analysis of the ratio of ipsilateral/contralateral hemisphere volume that treated with 10% sucrose, FE, PLGA, PLGA-FE, PLT@PLGA, PLT@PLGA-FE, RGD-PLT@PLGA, RGD-PLT@PLGA-FE. n = 6, 6, 7, 6, 6, 6, 6, 6 (from left to right). Data presented as mean ± SD. *, *p* < 0.05, **, *p* < 0.01, ***, *p* < 0.001

To test the effects of RGD-PLT@PLGA-FE on the outcomes of stroke, in vivo experiment was conducted following the experimental design illustrated in Fig. 4A. Neurobehavioral tests including modified neurological severity score (mNSS), hanging wire test and grid walking test were performed at different time points after tMCAO. Compared to the mice injected with 10% sucrose, the neurological deficits of mice injected with RGD-PLT@PLGA-FE were decreased. RGD-PLT@PLGA-FE treatment further decreased the neurological deficits compared to PLT@PLGA-FE treatment (Fig. 4B). Elevated body swing test showed that RGD-PLT@PLGA-FE treated mice swinged more balancedly than mice treated by 10% sucrose, but did not decrease neurological deficits compared with PLT@PLGA-FE (Fig. 4C). As evaluated by grid walking test (Fig. 4D) and hinging wire test (Fig. 4E), mice injected with RGD-PLT@PLGA-FE performed better than control mice at 14 days after stroke. The death rate of each group demonstrated no significant difference among the groups (Additional file 1: Table S1 and Fig S2).

As shown in Fig. 4F and G, the ratio of ipsilateral/contralateral hemisphere volume in RGD-PLT@PLGA-FE treated mice was 87.93 ± 7.40%, which was significantly higher comparing to that of 10% sucrose-treated, FE-treated, PLGA-treated, PLGA-FE-treated, PLT@PLGA-treated, and RGD-PLT@PLGA-treated mice, suggesting RGD-PLT@PLGA-FE treatment reduced brain atrophy volume of mice at 14 days after stroke.

The above results indicated that RGD modified-PLTs membrane coated PLGA nanoparticle could be used as a vehicle for targeted delivery of FE and thus continuously released FE to the ischemic brain area, which could further reduce the atrophy volume and improve neurobehavioral recovery of mice after stroke.

### RGD-PLT@PLGA-FE treatment improved angiogenesis and neurogenesis of ischemic mice

**Fig. 5:**
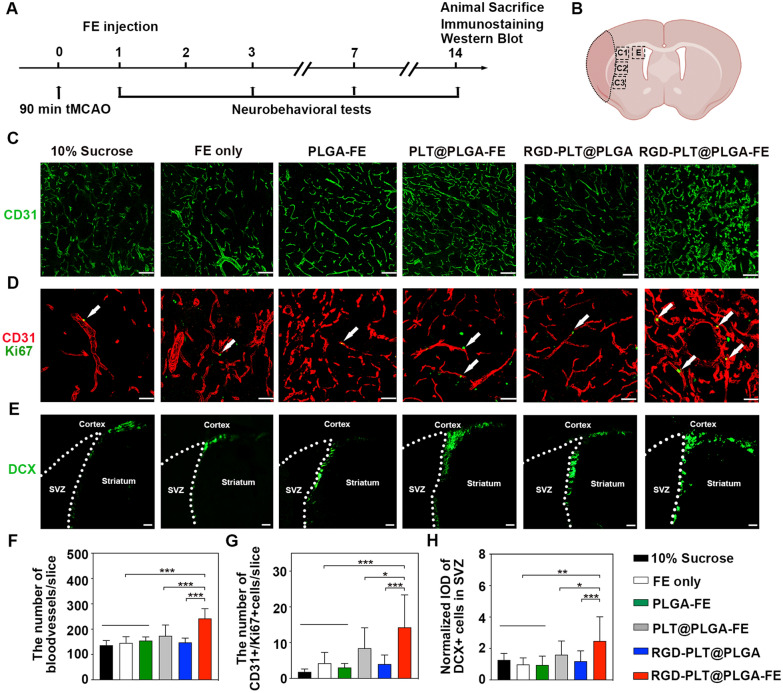
Immunohistochemical analysis of angiogenesis and neurogenesis of stroke mice. **A** Experimental scheme. **B** The pattern diagram of the peri-infarct region. Fluorescence imaging and quantitative analysis were performed in C1, C2, C3 and E. **C** Pictures showed CD31^+^ (green) microvessels in the peri-infarct region at 14 days after stroke. Scale Bar = 100 μm. **D** Pictures showed CD31^+^ (red) /Ki67^+^(green) cells in the peri-infarct region at 14 days after stroke. Scale Bar = 50 μm. **E** Pictures showed DCX^+^ (green) cells in the SVZ at 14 days after stroke. Scale Bar = 75 μm. **F** Quantitative analysis of the number of blood vessels of mice that treated with 10% sucrose, FE, PLGA-FE, PLT@PLGA-FE, RGD-PLT@PLGA, RGD-PLT@PLGA-FE at 14 days after stroke. n = 4, 4, 3, 4, 4, 4 (from left to right). **G** Quantitative analysis of the number of CD31^+^/Ki67^+^ cells of mice that treated with 10% sucrose, FE, PLGA-FE, PLT@PLGA-FE, RGD-PLT@PLGA, RGD-PLT@PLGA-FE at 14 days after stroke. n = 3/group. **H** Quantitative analysis of DCX^+^ cells in SVZ of mice that treated with 10% sucrose, FE, PLGA-FE, PLT@PLGA-FE, RGD-PLT@PLGA, RGD-PLT@PLGA-FE at 14 days after stroke. n = 4, 3, 3, 4, 4, 4 (from left to right). Data presented as mean ± SD. *p < 0.05, **p < 0.01, ***p < 0.001

Because our previous results showed that PLGA, PLT@PLGA and RGD-PLT@PLGA treatment showed similar effects on neurobehavioral recovery and brain atrophy volume, only RGD-PLT@PLGA treated mice were used as control in our following studies. To investigate the possible reason why RGD-PLT@PLGA-FE treatment achieved the best therapeutic effects for ischemic stroke, angiogenesis and neurogenesis of ischemic mice treated with 10% sucrose, FE only, PLGA-FE, PLT@PLGA-FE, RGD-PLT@PLGA, and RGD-PLT@PLGA-FE were examined by immunohistochemistry (Fig. 5A and B). As shown in Fig. 5C and D, the number of CD31^+^ blood vessels in the peri-lesion area of mice treated with RGD-PLT@PLGA-FE was increased compared to control mice, and mice treated with PLGA-FE or PLT@PLGA-FE. And meanwhile, the number of CD31^+^/Ki67^+^ endothelial cells were increased in RGD-PLT@PLGA-FE treated mice, suggesting only FE loaded with RGD modified PLTs membrane coated PLGA nanoparticles could achieve sufficient angiogenesis. We also found more DCX^+^ neuroblasts in the ischemic mice that treated with RGD-PLT@PLGA-FE (Fig. 5E), but not in other goups. These data suggested that injection of RGD-PLT@PLGA-FE increased angiogenesis and neurogenesis in stroke mice.

### RGD-PLT@PLGA-FE treatment increased cerebral blood flow of ischemic mice

**Fig. 6:**
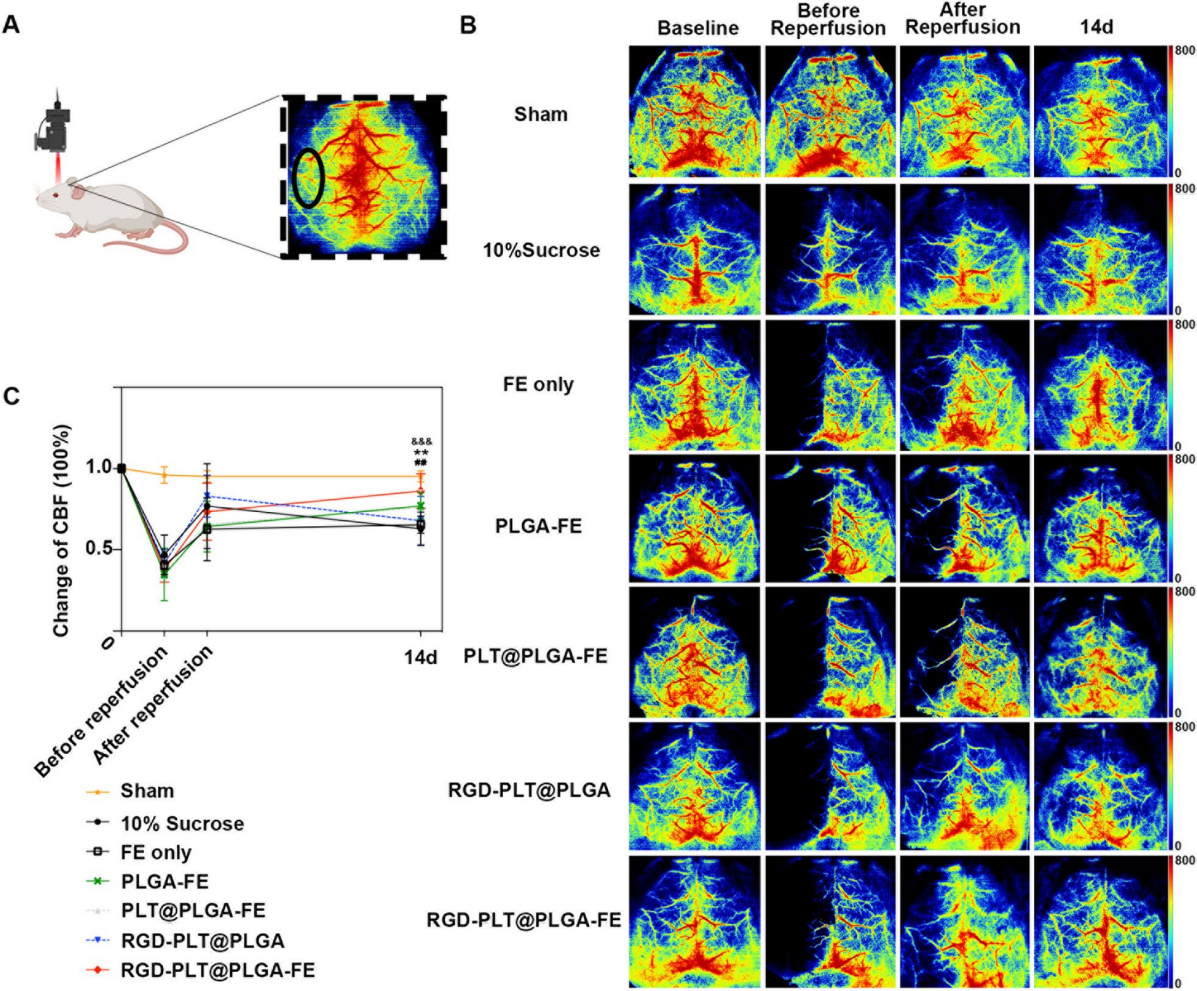
Changes in blood flow after ischemic stroke by laser sparkle imaging. **A** The pattern diagram of laser speckle imaging. Blood flow was calculated from the area indicated by the black circle. **B** The laser sparkle imaging of baseline, before reperfusion, after reperfusion and 14 days after stroke of mice treated with sham, 10% sucrose, FE, PLGA-FE, PLT@PLGA-FE, RGD-PLT@PLGA and RGD-PLT@PLGA-FE. **C** Quantitative analysis of the blood flow of seven groups including sham, 10% sucrose, FE, PLGA-FE, PLT@PLGA-FE, RGD-PLT@PLGA and RGD-PLT@PLGA-FE treated mice. &&&, RGD-PLT@PLGA-FE vs 10% Sucrose. **, RGD-PLT@PLGA-FE vs FE. ##, RGD-PLT@PLGA-FE vs RGD-PLT@PLGA. n = 8, 8, 7, 7, 6, 8, 7 (from left to right). Data presented as mean ± SD. **, ##*p* < 0.01, &&&*p* < 0.001

To further explore if RGD-PLT@PLGA-FE treatment promotes cerebral blood flow of stroke mice, laser speckle imaging was used to measure the cerebral blood flow in sham mice and ischemic areas of stroke mice that treated with 10% sucrose, FE only, PLGA-FE, PLT@PLGA-FE, RGD-PLT@PLGA, and RGD-PLT@PLGA-FE. As shown in Fig. 6A, cerebral blood flow was measured before surgery, before reperfusion, immediately after reperfusion, and 14 days after stroke. The color-coded images and quantitative analysis of blood flow in Fig. 6B and C showed that RGD-PLT@PLGA-FE administration increased the blood flow in the ischemic areas at 14 days after stroke, compared to other groups.

### RGD-PLT@PLGA-FE treatment promoted the expression of neurotrophic factors

**Fig. 7:**
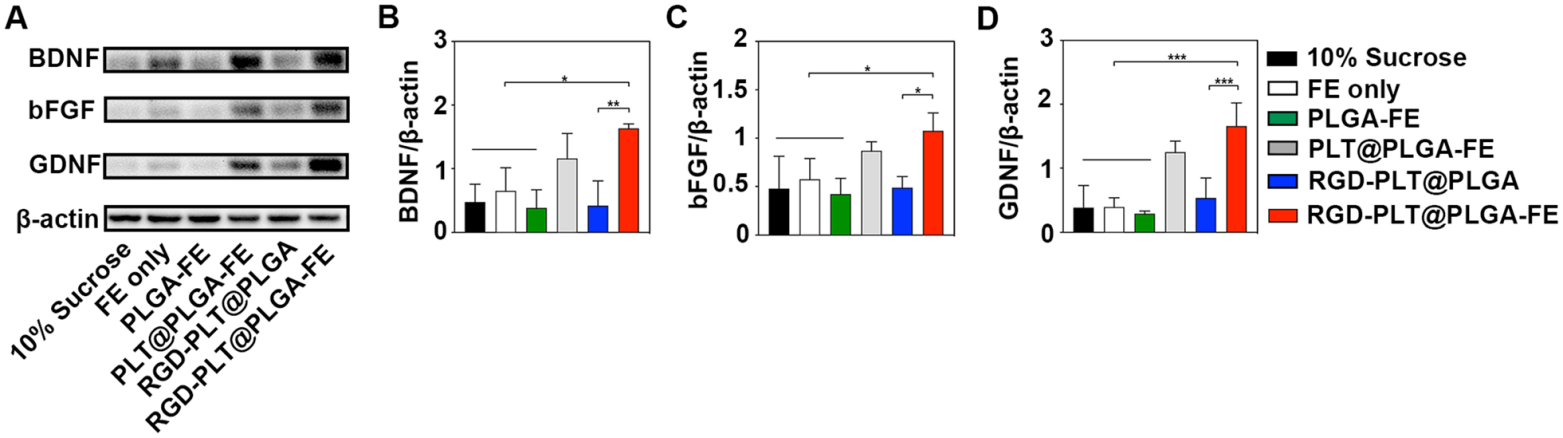
Expression of BDNF, bFGF and GFND in stroke mice brain. **A** Western blotting analysis of neurotrophic factors including BDNF, bFGF, GDNF at 14 days after stroke. **B** Quantitative analysis of BDNF/β-actin at 14 days after stroke. n = 3/group. **C** Quantitative analysis of bFGF/β-actin at 14 days after stroke. n = 3/group. **D** Quantitative analysis of GDNF/β-actin at 14 days after stroke. n = 3/group. Data presented as mean ± SD. *p < 0.05, **p < 0.01, ***p < 0.001

To further explore the molecular mechanism of RGD-PLT@PLGA-FE induced angiogenesis and neurogenesis in stroke mice, western blot was used to quantify the expression of growth factor including BDNF, bFGF, and GDNF. The results indicated that RGD-PLT@PLGA-FE treatment increased the expression of BDNF, bFGF, and GDNF (Fig. 7A–D), suggesting that FE released from RGD-PLT@PLGA-FE into mouse brain contains BDNF, bFGF, and GDNF.

### In vivo biological safety of RGD-PLT@PLGA-FE

**Fig. 8:**
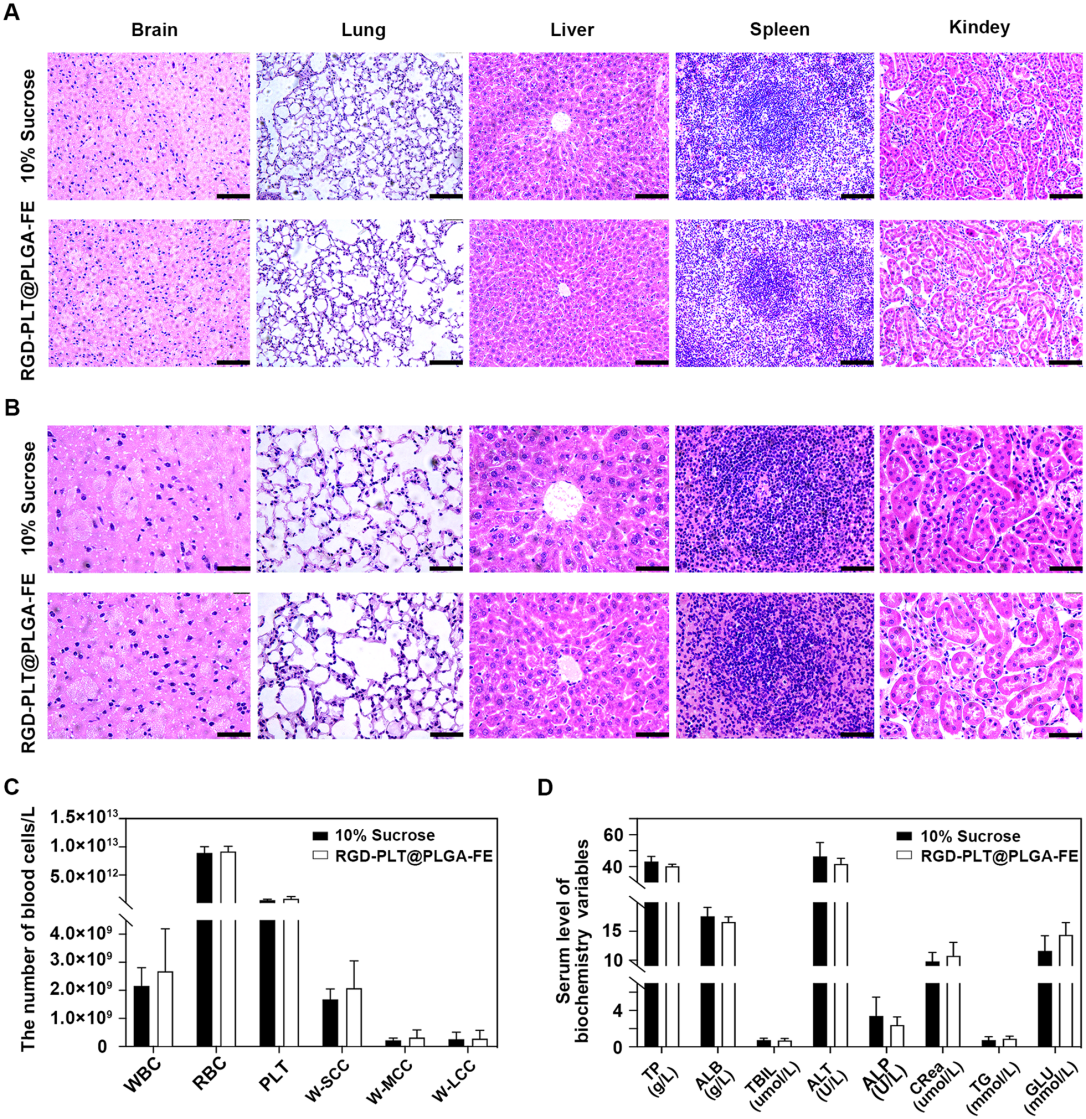
In vivo biocompatibility of RGD-PLT@PLGA-FE. **A** H&E staining of main organs including brain, lung, liver, spleen, kidney in 10% sucrose and RGD-PLT@PLGA-FE treated mice. Scale Bar = 100 μm. n = 5/group. **B** H&E staining of main organs including brain, lung, liver, spleen, kidney in 10% sucrose and RGD-PLT@PLGA-FE treated mice. Scale Bar = 50 μm. n = 5/group. **C** Quantitative analysis of blood routine tests in 10% sucrose and RGD-PLT@PLGA-FE treated mice. n = 5/group. **D** The analysis of serum blood chemistry test in 10% sucrose and RGD-PLT@PLGA-FE treated mice. n = 5/group. Data presented as mean ± SD

The safety of nanoparticles in vivo is an important criterion for clinical translation. To investigate the safety of RGD-PLT@PLGA-FE in vivo, RGD-PLT@PLGA-FE was injected into healthy mice through tail vein at the dose of 200 mg/kg of nanoparticles (30 mg/ml). After treatment with 10% sucrose and RGD-PLT@PLGA-FE, H&E staining of the tissue samples including brain, lung, liver, spleen, and kidney were conducted. The results showed that there were no abnormal inflammatory cell infiltration and changes in tissue structure (Fig. 8A and B), suggesting the nanoparticles we developed in our study presenting great biocaompatibility. Then blood routine test and serum blood biochemical test were performed to monitor potential systemic toxicity. We found that 2 days after administration, the number of WBC, RBC, PLT, W-SCC, W-MCC, W-LCC, and the serum level of TP, ALB, TBIL, ALT, ALP, CRea, TG and GLU were no difference between 10% sucrose and RGD-PLT@PLGA-FE groups (Fig. 8C and D).

## Discussion

We have previously reported that cell-free extract from human adipose tissue showed proangiogenic effect in a murine model of limb ischemia [17], as well as improved fat graft survival [18]. However, high doses of FE are required to inject repeatedly to achieve a satisfied therapeutic outcome, which often considered as one of the bottlenecks from bench to bedside. In the study, we developed an RGD conjugated and PLTs membrane coated PLGA nanoparticle platform. Results demonstrated that RGD-PLT@PLGA-FE could efficiently and targeted deliver FE into the lesion area of the brain, and a single injection of nanoparticles could suffucuently increase blood flow and neurobehavioral recovery through angiogenesis and neurogenesis, without inducing detectable adverse effects. Overall, our study suggested the clinical translational potential of RGD-PLT@PLGA-FE for stroke treatment.

Coating nanoparticles with natural cell membranes has been recognized as an attractive strategy in nanotherapeutics because of their unique properties such as negligible immunogenicity, long blood circulation, and inherited specific molecular recognition[26]. Nanoparticles coated with PLTs membranes have received wide attention as PLTs exhibit a prolonged half-life of approximately 30 h, which is attributable to its surface marker CD47, a protein that interacts with the signal regulatory protein α (SIRPα) on the immune cells and therefore inhibits the immune clearance[28–30]. Besides that, PLTs also express many receptors that could specifically adhere to damaged and inflamed vasculature[31]. In addition, a peptide sequence of Arg-Gly-Asp (RGD) motif has been demonstrated to target and adhere toward molecules (α6β1, α5β1, α2β1, αvβ3, and αIIbβ3)[32] that high expressed on endothelial cells, especially RGD shows high affinity to αvβ3 integrin that expressed on the surface of angiogenic blood vessels[33]. Thus, we conjugated RGD peptide to the PLTs membrane to synergistically enhance its ability to target damaged and angiogenic blood vessels. As a result, particles coated by RGD-PLT improved their accumulation and targeting in ischemic mice, while fewer PLT@PLGA and PLGA were accumulated in the brain, indicating the benefits were obtained from both PLTs coating and RGD modification in the current system. In our research, we found more RGD-PLT@PLGA accumulated in lung comparing with bare PLGA in lung. Bare PLGA nanoparticle aggregated quickly up to micro-scale when transferred into 50% FBS due to the electrostatic shielding effect, which resulted in the major biodistribution of bare PLGA nanoparticle in reticuloendothelial system (RES) organs (including liver and spleen) but not in lung after i.v. injection. However, PLT membrane coating inhibited such aggregation of PLGA nanoparticle in 50% FBS, and prolonged their circulation time in blood, which resulted in wide distribution of RGD-PLT@PLGA in liver, spleen, lung, kidney, and bloodstream, but rather in the liver. This distribution behavior of RGD-PLT@PLGA was consistent to other cellular membrane-coated PLGA nanoparticle verified in previous published studies [34, 35]. RGD-PLT@PLGA nanoparticles are good delivery systems for other proteins or drugs, and could be applied in many diseases, which is supported by previous study. Based on the properties of self-recognizing of RGD and platelet, RGD-PLT@PLGA nanoparticles also could be applied in tumor therapy. Recently, Jing et al. developed RGD-PLT@PLGA nanoparticles as drug delivery system loaded with melanin nanoparticles (MNPs) and doxorubicin (DOX) to inhibit tumor growth and metastasis by photothermal therapy [36]. Therefore, RGD-PLT@PLGA nanoparticles can be used in other diseases.

The double emulsion method that is used to load hydrophilic proteins is a common preparation technology. While the toxic organic solvent dichloromethane involved in the preparation of PLGA-FE may have some side effects on the protein compostions of FE. Our research indicated that protein profiles released from PLGA-FE exhibited similar to free FE and growth factors including GDNF, bFGF, TGF-β and VEGF in PLGA-FE displayed comparable levels to those in free FE, suggesting PLGA-FE fabrication did not affect the protein profiles of FE. In addition, part of the weak adsorption protein would accumulate ulate on the surface of PLGA by the double emulsion method [37], which results in the burst release phenomenon. To address this issue, we performed the same process but added three washes following the synthesis of RGD-PLT@PLGA-FE, thus the weak adsorption or superficial protein would be removed. a controlled release of protein from RGD-PLT@PLGA-FE, rather than a burst release manner was detected.

At present, only tPA and thrombectomy have been proven effective for treating ischemic stroke in clinic [38, 39]. However, the therapeutic time window and risk of hemorrhage greatly limited the clinical applications. In addition, more than 1500 neuroprotective drugs that show dramatic efficacy in the experimental animal models of ischemic stroke but failed in clinic trial [40]. Recently, neurorestorative-based therapy has attracted large attention [41]. Angiogenesis and neurogenesis are two key processes for neurorepair after stroke [42]. Newly formed capillaries could supply oxygen and nutrition to the ischemic region for enhancing tissue repairing and remodeling, and newly formed neuroblasts could migrate to the damaged area, where they become matured neurons to replace the dead neurons [43]. Our in vitro study showed that incubation of HUVECs with fat extract (FE), PLGA-FE, PLT@PLGA-FE or RGD-PLT@PLGA-FE increased the tube formation and migration of HUVECs, while PLGA only, PLT@PLGA, RGD-PLT@PLGA showed useless, suggesting that PLGA nanoparticles do not have any benefits for angiogenesis. Our in vivo study found that single injection of RGD-PLT@PLGA-FE into ischemic mice model could sufficiently increase angiogenesis and neurogenesis, as well as increase blood flow, while the effect of PLGA itself in angiogenesis and neurogenesis is negligible. In addition, only one-time injection of FE did not affect angiogenesis and neurogenesis, this is probably due to the short half-life of FE and lack of targeting capability, and further highlighted the important strategy of loading FE into PLGA-based nanoparticles.

After ischemic stroke, vasculature is damaged in the core region of ischemic brain, however, in the peri-lesion area angiogenesis will occur at the subacute stage of stroke [44]. It has been reported that a positive correlation was observed between patient survival and density of angiogenic vessels. In our study we used RGD peptides decorated PLT membrane coated nanoparticle as a delivery vehicle to targeted deliver FE to angiogenic vessels, which is important to further increase angiogenesis in the peri-lesion area and neurobehavioral recovery of stroke mice.

The effect of RGD-PLT@PLGA-FE in angiogenesis and neurogenesis is mainly due to high amount of growth and neurotrophic factors in FE. Our previously studies demonstrated that FE is enriched in several high levels of growth factors, including BDNF, GDNF, TGF-beta, and bFGF etc. [17, 18]. These factors are important modulators during angiogenesis and neurogenesis. We found that FE contains high levels of growth factors, including BDNF, GDNF, TGF-β, HGF, bFGF, etc. In addition, our proteomic analysis revealed that 56 proteins were involved in angiogenesis [17]. Cell proliferation, migration and tube formation of HUVECs were reduced by proteinase K treatment, suggesting that those proteins in FE were essential for the observed proangiogenic activity[18]. Our study also demonstrated that injection of RGD-PLT@PLGA-FE delivered BDNF, bFGF and GDNF to mice brain after stroke, which could contribute to angiogenesis and neurogenesis, and subsequently improved neurobehavioral recovery.

Both TTC staining and cresyl violet staining are commonly used for evaluating ischemic stroke. TTC staining is routinely used to distinguish the infarct area and penumbra area. But a large number of studies provide evidence that penumbra region exists for 7 h, and probably for as long as 72 h [45, 46]. In our study we mainly focus on the effects of RGD-PLT@PLGA-FE at 14 days after stroke, the chronic stage of stroke. Penumbra area has already been disappeared at 7 days after stroke (Additional file 1: Fig S8). As the main focus of our study is to evaluate the effects of RGD-PLT@PLGA-FE on brain atrophy, angiogenesis and neurogenesis at the chronic stage of stroke, cresyl violet staining is similar to TTC staining.

One limitation of laser speckle imaging is the depth is relatively low, and blood flow could only be detected on the surface of cortex. In our study, the purpose of using laser speckle imaging is to evaluate the effects of RGD-PLT@PLGA-FE on blood flow. We acknowledge that the blood flow detected by laser speckle imaging is mainly from the cortex, however, we still found RGD-PLT@PLGA-FE treatment improved blood flow, compared to other groups. In future, we may need to perform magnetic resonance angiography (MRA) or synchrotron radiation angiography to measure the blood flow of deep layer of brain.

For biomedical applications of biomaterials, biosafety is an important evaluation index. PLGA has provided a safe and non-toxic building block for various control-release systems as a biocompatible and biodegradable polymer [47, 48]. In our work we did not detect noticeable side-effects such as inflammation, swelling in the brain, lung, liver, spleen, and kidney after exposure of PLGA-based nanoparticles. What’s more, the biochemical analysis of blood and serum suggested the particles we used are not cytotoxic. Previous study reported that PEG coated PLGA can increase the circulation time of nanoparticles in vivo, but one of the problems is the entrapment of nanoparticles in the mononuclear phagocytic system as present in the liver and spleen [49]. In our study we did not find any side effects in RGD-PLT@PLGA treated mice, accompanied with decreased cellular capture by RES. One limitation of our study is that the observation window is relatively short, whether RGD-PLT@PLGA is safe in long time needs further investigation.

## Conclusions

In our study, we developed a RGD modified PLTs membrane cloaked nanoparticle for targeted deliver fat extract (FE) and demonstrated that RGD-PLT@PLGA-FE promoted neurobehavioral recovery of ischemic stroke via increasing angiogenesis and neurogenesis, indicating the potential for treatment of stroke.

## Methods

### Conjugation of platelet membrane with RGD peptide

The whole blood was collected from adult ICR male mice via submandibular puncture with an EDTA-coated tube (BD Microtainer™, BD, Franklin Lakes, NJ). After centrifugation at 300 ×*g* for 5 min, the resultant supernatant was collected and subjected to 300 ×*g* centrifugation for another 5 min. Following that, the supernatants were centrifuged at 2000 ×*g* for 10 min and platelet enriched precipitation was resuspended in water. After that, the PLT membrane was collected through repeated freeze–thaw cycles. Briefly, the purified platelet in water was quickly frozen at − 80 °C and then thawed at room temperature (RT). After completely thawed, the platelets were centrifuged at 25,000 ×*g* for 10 min, and the pellet was resuspended in the water again. The platelet suspension was then frozen at − 80 °C, and the process was repeated three times. After the last cycle, the pellet was resuspended in water and the membrane protein concentration was determined by bicinchoninic acid assay (BCA, Thermo Fisher Scientific, Waltham, MA). The RGD-conjugated PLT membrane (RGD-PLT) was prepared by a lipid-insertion method. Briefly, Arginine-glycine-aspartic acid-tyrosine-cysteine (RGDyC, Jiangsu Ji Tai Peptide Industry Science and Technology, Jiangsu, China) containing cysteine group was firstly reacted with maleimide group in DSPE-PEG2000-Maleimide (Avanti Polar Lipid, Merck KGaA, Darmstadt, Germany) to form the DSPE-PEG2000-RGD at a molar ratio of 1: 1. The synthesized DSPE-PEG2000-RGD was then mixed with PLT membrane in a fixed ratio of 5% (w/w) to obtain RGD-platelet (RGD-PLT) spontaneously.

### Preparation of FE-loaded PLGA nanoparticles

FE was loaded into PLGA nanoparticles by a double emulsion method. PLGA (0.67 dl/g carboxy-terminated 50: 50 poly (lactic-co-glycolic) acid, LACTEL Absorbable Polymers, Birmingham, AL) was dissolved in dichloromethane at 60 mg/ml. Meanwhile, fat extract (FE) was obtained from healthy female donors according to previously published work [17] that was approved by Ethics Committee of Shanghai Jiao Tong University. Then, 1 ml of polymer was added to 100 μl of FE at various protein concentrations and subjected to sonication (150E Sonic Dismembrator, Thermo Fisher Scientific, Waltham, MA) following the parameters (85% power with a pulse of 2 s on/2 s off) for 5 min. The obtained 500 μl first-emulsion suspension was then added into a 5 ml outer aqueous phase that contained 0.7% polyvinyl alcohol (PVA, Sigma-Aldrich, Saint Louis, MO) (w/v) in water and sonicated using the same setting for another 5 min. The emulsion was then added to 25 ml water with a stirring speed at 200 rpm for 2.5 h in the ice bath. After stirring, the particles (PLGA-FE) were pelleted by centrifugation at 25,000 × g for 10 min, and the supernatant was collected for protein measurements by BCA. Encapsulation efficiency (EE%) was calculated by (total protein input – free protein in supernatant) divided by the total protein added. Loading capacity (LC%) was calculated by (total protein added—free protein in the supernatant) divided by the total weight of PLGA plus entrapped protein. As a result, the initial input of FE at 30 mg/ml was determined as an optimal concentration for FE encapsulation by PLGA nanoparticle (PLGA-FE), and then used for PLT membrane coating. The bare PLGA (PLGA) without FE encapsulation was synthesized according to the same inner phase process by adding 1 ml of polymer into 100 μl of water and followed by the same emulsion process. Furthermore, the lipophilic dyes (3,3′-dioctadecyloxa carbocyanine perchlorate (DiO) and 1,1′-dioctadecyl-3,3,3′,3′-tetramethylindodicarbo cyanine perchlorate (DiD)) labeled PLGA nanoparticles were prepared by adding 1 ml of polymer that consisting of 0.2 wt% DiO or DiD into 100 μL of water and followed by the same emulsion procedure.

### Preparation and characterization of RGD-PLT@PLGA-FE nanoparticles

PLGA-FE was mixed with RGD-PLT at a ratio of PLGA mass to PLT membrane protein at 2: 1 (w/w) and sonicated by a water sonicator (FB120, Fisherbrand, Ottawa, Canada) for 3 min to prepare the RGD-PLT@PLGA-FE. This formulation was then suspended in 10% sucrose in water and stored at − 80 °C for further use. PLT@PLGA-FE, RGD-PLT@PLGA, and PLT@PLGA, were synthesized following the same ratio (w/w) of PLGA mass (regardless of FE loading or not) to PLT membrane protein (regardless of RGD conjugation or not).

To visualize the RGD-PLT@PLGA-FE, sample suspension at 0.5 mg/ml (PLT membrane protein) was dropped onto the carbon-coated Cu grids and stained with 1% uranyl acetate. After drying, the morphological images of RGD-PLT@PLGA-FE were taken with a TEM (JEM-2010, JEOL, Tokyo, Japan) at 200 kV acceleration voltage. Hydrodynamic diameter and superficial charge of RGD-PLT@PLGA-FE were tested by DLS (Nano Zetasizer, Malvern, Malvern city, UK). To evaluate the stability of RGD-PLT@PLGA-FE, hydrodynamic diameter of sample was test in water, 1 × PBS and 50% FBS (v/v) solution over 0 d, 1 d, 3 d, 5 d, 7 d by DLS (Nano Zetasizer, Malvern, Malvern city, UK). The hydrodynamic diameter of bare PLGA and RGD-PLT@PLGA-FE was examined in water and in 50% FBS (v/v) solution by DLS (Nano Zetasizer, Malvern, Malvern city, UK). Subsequently, the hydrodynamic diameter of bare PLGA was detected in 50% FBS (v/v) solution over 0 d, 1 d, 3 d by DLS (Nano Zetasizer, Malvern, Malvern city, UK).

To study the protein compositions in the RGD-PLT@PLGA, sodium dodecyl sulfate–polyacrylamide gel electrophoresis (SDS-PAGE) and western blot (Epizyme, Shanghai, China) were performed. PLT vesicles (PLT ghost), PLT@PLGA, and RGD-PLT@PLGA were freshly prepared and proteins in those samples were extracted respectively with a RIPA lysis buffer (Millipore, Billerica, MA). All samples were then mixed with SDS-PAGE loading buffer and heated at 95 °C for 5 min. After that, samples were loaded into the SDS-PAGE gel for electrophoresis analysis. Following electrophoresis, proteins in all samples were stained with Coomassie blue and detained in water before imaging. For western blot, all proteins were translocated onto the PVDF membrane (Millipore, Billerica, MA) at 300 mA for 90 min. After blocking the membrane with 10% protein-free protein blocking solution (Epizyme, Shanghai, China), the membrane was immunostained with CD62p (1:1000 dilution, ab255822, Abcam, Cambridge, UK), and CD41(1:1000 dilution, ab134131, Abcam, Cambridge, UK) primary antibodies. After washing with TBS containing 0.05% Tween 20 twice, the membrane was incubated with horseradish peroxidase (HRP)-conjugated secondary antibody (Invitrogen, Carlsbad, CA) for another 1 h. After the addition of ECL western blot substrate (Meilunbio, Dalian, China), blot signals were visualized and analyzed by an imaging system (ChemiDoc ™ XRS + , Bio-Rad, Hercules, CA).

To test the FE release, RGD-PLT@PLGA-FE in PBS after synthesis was immediately aliquoted into seven equal volumes and placed in a 37 °C incubator with a tube rotator (Scilogex, Lexington, MA). At the intended time points (0 h, 0.1 h, 12 h, 24 h, 48 h, 72 h, 120 h and 168 h) after incubation, the samples were centrifuged at 25,000 ×*g* for 10 min and the supernatant was collected for protein quantification using QuantiPro^TM^BCA (Sigma-Aldrich, Saint Louis, MO) followed the vendor’s instructions. To remove the weak adsorption or superficial protein in RGD-PLT@PLGA-FE, the same process was performed and three more washes were added following the synthesis of RGD-PLT@PLGA-FE. Afterward, FE released from RGD-PLT@PLGA-FE at various predetermined time points (0 h, 0.1 h, 12 h, 24 h, 48 h, 72 h, 120 h and 168 h) was collected for protein quantification using QuantiPro^TM^BCA (Sigma-Aldrich, Saint Louis, MO) following the previous protocol.

To analyze the protein compositions of FE before and after dichloromethane treatment in the process of encapsulation, SDS-PAGE and ELISA assay were performed. Free FE and FE released from PLGA-FE for 24 h were collected at an equivalent protein concentration. All samples were then mixed with SDS-PAGE loading buffer and heated at 95 °C for 5 min. After that, samples were loaded into the SDS-PAGE gel for electrophoresis analysis. Following electrophoresis, proteins in all samples were stained with Coomassie blue and detained in water before imaging. For ELISA assay, the levels of multiple key proteins including GDNF (E-EL-H1495c, Elabscience, Wuhan, China), bFGF (E-EL-H6042, Elabscience, Wuhan, China), TGF-β (E-EL-0162c, Elabscience, Wuhan, China) and VEGF (E-TSEL-H0026, Elabscience, Wuhan, China) in FE either before or after encapsulation at equivalent protein ratios were quantified. Fifty microliters samples added and incubated at 37 °C for 90 min. The plates were washed and added 100 µl each enzyme conjugate working solution in an incubator at 37 °C for 30 min. The plates were washed again and incubated with 90 µl substrate solution (TMB) in each well at 37 °C for 15 min in the dark room. Fifty microliters each terminate fluid was added and immediately measured using a plate reader (SpectraMax I3, MD, San Jose, CA) at 450 nm.

Conjugation efficiency of RGD to DSPE-PEG2000-Maleimide was determined by HPLC. Briefly, 1 mg/ml of RGD dissolved in water was run by HPLC following the parameters as below: A solution was 100% acetonitrile containing 0.1% trifluoroacetic acid, and B solution was 100% ddH_2_O containing 0.1% trifluoroacetic acid; the column was equilibrated with a mobile phase of A/B at 2/98 (v/v) over 30 min with a flow rate of 0.7 ml/min; the detection wavelength was at 220 nm. Meanwhile, 1 mg/ml of RGD either as free form (filtered free RGD) or preincubation with DSPE-PEG2000-Maleimide (filtered after RGD conjugation) for 30 min at 37 °C were then filtered by ultrafiltration (molecular weight cutoff = 3 kDa; Millipore, Billerica, MA), and the resulting filtered out solutions were measured by HPLC. Percentage of initial free RGD minus filtered out percentage of free RGD is the conjugation efficiency of RGD to DSPE-PEG2000-Maleimide.

To verify whether RGD was inserted into the PLT membrane on the nanoparticles, (FITC) RGD-PLT was synthesized through a lipid-insertion method. Briefly, DSPE-PEG2000-Maleimide with FITC labelling RGDyC at a molar ratio of 1 to 1 was mixed in water at 37 °C for 60 min to form DSPE-PEG2000-RGD(FITC). After incubation, PLT membrane was prepared, followed by the addition of DSPE-PEG2000-RGD(FITC) at a 5% lipid ratio (w/w) and incubation at 37 °C for 60 min. Afterwards, the sample was washed with 1 × PBS for 3 times and dispersed in water. Then, the obtained (FITC)RGD-PLT was mixed with PLGA core at a ratio of PLT membrane protein to PLGA mass of 1: 2 (w/w) and sonicated for 3 min to prepare (FITC)RGD-PLT@PLGA. The fluorescence of (FITC)RGD-PLT@PLGA was measured using a plate reader (SpectraMax I3, MD, San Jose, CA) at an excitation/emission of 488/525 nm. The percentage of conjugated (FITC)RGDyC onto nanoparticle was calculated by dividing the initial input. Free (FITC)RGD with various concentrations were dissolved in water to make the fluorescent standard curve. The DSPE-PEG2000-Maleimide conjugated with RGDyC without FITC labelling (denoted RGD-PLT@PLGA) was used as a negative control. (FITC)RGDyC directly mixed with PLT, followed by washing 3 time with PBS and sonicated with PLGA (denoted (FITC)RGD + PLT@PLGA), was used as another negative control.

### Cell culture and preparation

The cells are HVUVEC cells (DFSC-EC-01, Zhong Qiao Xin Zhou Biotechnology, Shanghai, China), and ECM culture medium (Zhong Qiao Xin Zhou Biotechnology, Shanghai, China, Catalogue number: ZQ-1304) contains 5% FBS, 1% cell growth factors and 1% penicillin/streptomycin solution. Cells were cultured in an incubator at 37 °C with 5% CO_2_.

### Tube formation

PLGA, PLGA-FE, PLT@PLGA, PLT@PLGA-FE, RGD-PLT@PLGA, and RGD-PLT@PLGA-FE (15 mg/ml each) were placed in the blank culture medium at 4 °C for 12 h, then the culture medium was centrifuged to collect the supernatant, and treated HUVECs (2 × 10^4^ cells of each well) in 96-well plates at 37 °C with 5% CO_2_ for 6 h. 24 µg FE was added to HUVECs as a positive control. After that, HUVECs were observed and photographed with an inverted microscope (TCS SP5, Leica, Wetzlar, Germany). One field of each well was randomly selected and imaged and 3 independent trials were performed. The number of branches of tubes was counted with Image J software (V 1.53).

### Migration test

HUVECs (1.2 × 10^5^ cells of each well) were cultured in 24-well plate. When cells proliferated to a density of 90%, mitomycin was used to treat the cells for 3 h to inhibit proliferation. Then each well was washed by 1 × PBS for 3 times. 200ul-pipette tip scratched on the plate. PLGA, PLGA-FE, PLT@PLGA, PLT@PLGA-FE, RGD-PLT@PLGA, and RGD-PLT@PLGA-FE (15 mg/ml each) were placed in the blank culture medium at 4 °C for 12 h, then the culture medium was centrifuged to collect the supernatant, and treated HUVECs in a 37 °C, 5% CO2 incubator for 24 h and observed and photographed with an inverted microscope (TCS SP5, Leica, Wetzlar, Germany). Each group had 3 independent trial and each trial randomly selected one field of well plate. The scratch areas (SA) were calculated at 0 h and 24 h, and change in scratch areas (ΔSA) was calculated using the following formula with Image J software.

$$\mathrm{\Delta SA}= \frac{{SA}_{0h}-{SA}_{24h}}{{SA}_{0h}}$$

where ${SA}_{0h}$ represents the initial scratch areas and ${SA}_{24h}$ is the scratch areas at 24 h after scratch formation.

### Aggregation assay of RGD-PLT@PLGA

Inflamed HUVECs was established by LPS (2 µg/ml) treatment for 24 h. Each well was washed with 1 × PBS for 3 times. Then HUVECs were treated with DiO labeled PLGA, DiO labeled PLT@PLGA, or DiO labeled RGD-PLT@PLGA (4 mg/ml each) for 2 h. Then each well was washed with 1 × PBS for 3 times. Cells were then observed under the confocal microscope (TCS SP5, Leica, Wetzlar, Germany), or digested by 0.25% trypsin and examined by flow cytometry (FACSAria II, BD, Franklin Lakes, NJ). Non-treated HUVECs were used as a negative control.

### Cell viability and cell proliferation

HUVECs (2 × 10^4^ cells of each well) were cultured in 96-well plates at 37 °C with 5% CO_2_ for 6 h. PLGA, PLGA-FE, PLT@PLGA, PLT@PLGA-FE, RGD-PLT@PLGA, and RGD-PLT@PLGA-FE (15 mg/ml each) were placed in the blank culture medium at 4 °C for 12 h, then the culture medium was centrifuged to collect the supernatant, and treated HUVECs in a 37 °C, 5% CO2 incubator for 18 h. 24 µg FE was added to HUVECs as a positive control. The OD value was detected in 24 h, 48 h, 72 h and 96 h for 450 nm.

### Transient middle cerebral artery occlusion

Transient middle cerebral artery occlusion (tMCAO) was performed as described previously [50]. The mice were anesthetized with isoflurane (1.5%-2%) in a mixture of oxygen/nitrous oxide (30%/70%) on the heating pad (RWD, Shanghai, China). External carotid artery (ECA) and internal carotid artery (ICA) were separated carefully. Then a 6–0 suture was inserted into the ICA approximately 10 mm through the ECA to occlude the middle cerebral artery (MCA). After 90 min, the suture was withdrawn from ICA for reperfusion. Successful occlusion of the MCA was evaluated by laser Doppler flowmetry (VMS, Moor, Wilmington, DE). Regional blood flow decreased by 80% compared to baseline after ischemia and recovered to 80% of baseline after reperfusion is considered as a successful model. The death rate of mice after tMCAO was recorded.

### Fluorescence imaging

One day after tMCAO model, 10% sucrose, DiD-labeled PLGA, DiD-labeled PLT@PLGA, and DiD-labeled RGD-PLT@PLGA (10 mg/ml, 125 µl each) were injected by tail vein. Twenty-four hours later, brain, heart, lung, liver, spleen, and kidney were dissected and measured by fluorescent imaging machine (IVIS® Lumina III, PerkinElmer, Waltham, MA) at an Excitation/Emission of 630/670 nm.

### Neurobehavioral tests

Twenty-four hours after tMCAO, 10% sucrose, PLGA, PLT@PLGA, RGD-PLT@PLGA, FE, PLGA-FE, PLT@PLGA-FE or RGD-PLT@PLGA-FE (30 mg/ml, 200 µl each) were injected into mice. Neurobehavioral tests were judged by another person blindly to animal’s groups. The tests were carried out at 1 d, 3 d, 7 d, 14 d after tMCAO. These neurobehavior tests mainly contain neurological severity score (mNSS), elevated body swing test, grid walking test and hanging wire test.

### Modified neurological deficit score

mNSS score is mainly a comprehensive assessment of neurofunction including motor, sensory and balancing function which were graded on a scale of 0 to 14 [51].

### Elevated body swing test (EBST)

EBST was carried out by raising the mice by its tail and observing the turns of the mice. A total of 20 repeats were performed. The result was number of deflection to the right/ total numbers. Results of the experiment were recorded.

### Grid walking test

Grid walking test was used to evaluate the motor coordination deficits after ischemic stroke. The mice were placed on an elevated square grid with each grid size was 1.5 cm × 1.5 cm. Mice were allowed to be familiar with the grid for 1 min. A camera was placed at the bottom of the grid to record the number of foot faults. Foot faults means mice could not walk accurately with their limbs on the grid. 75% ethanol was used to clean the grid after each trial. The statistical data is ipsilateral foot faults/(ipsilateral foot faults + contralateral foot faults).

### Hanging wire test

Hanging wire test aims to evaluate the upper limb strength and coordination of mice. Once mice hanged on the middle of the wire, timer was started. The initial score of each mouse was 10 points. When mice fell from the wire, the “falling score” would be declined by 1. Not stop until “falling score” is equal to 0 or costs 180 s. A dropping curve was drawn.

### Atrophy volume measurement

Mice were sacrificed on day 14 of tMCAO by cardiac perfusion. Firstly, 1 × PBS was perfused and then followed by 4% paraformaldehyde. Brain was postfixed in 4% paraformaldehyde for 2 h and dehydrated in 30% sucrose solution for 2 days. After that, brain was frozen in -80 ℃ for one day, cut into 30 µm sections, and stored in antifreeze solution. The sections were used for nissl staining. Following formula was used for statistical analysis of staining results.

$$V=\sum_{1}^{n}\frac{H[\mathrm{\Delta Sn}+\mathrm{\Delta S}\left(\mathrm{n}+1\right)+\sqrt{\mathrm{\Delta Sn}+\mathrm{\Delta S}\left(\mathrm{n}+1\right)}]}{3}$$

*H* is the distance of two adjacent sections (*H* = 300 μm) and ΔSn and ΔSn + 1 were the atrophy areas of two adjacent sections.

Mice were sacrificed on day 1 and day 7 of tMCAO (Sigma-Aldrich, Saint Louis, MO). After decapitation, the brain was removed and sectioned into 1 mm thick slice then immersed for 20 min into 2% 2,3,5-triphenyltetrazolium chloride (TTC, Sigma-Aldrich, Saint Louis, MO) at 37 ℃.

### Laser speckle imaging

The mice were anesthetized with isoflurane and fixed on the stereotaxic apparatus. Before tMCAO, before reperfusion, immediately after reperfusion and 14 days after stroke, the cerebral blood flow of the mouse was measured with a laser speckle imaging machine (RFLSI III, RWD, Shanghai, China). The exposure time is 2 ms, the shooting time is 3 s, frame rate is 10 frames/second, the magnification is 3 times, and the laser intensity is 110 mW. The average blood flow was measured on the left of frontal suture and calculated the change rate base on the blood flow before tMCAO.

### Immunofluorescence staining

The sections were taken out from the antifreeze solution and washed in 1 × PBS, incubated with 0.3% Triton X-100 for 10 min, and then incubated with 5% BSA to block for 1 h. The specific primary antibodies goat-anti CD31 (1:200 dilution, AF3628, R&D, Minneapolis, MN), rabbit-anti Ki67 (1:200 dilution, ab15580, Abcam, Cambridge, UK), and rabbit-anti DCX (1:200 dilution, ab18723, Abcam, Cambridge, UK) were incubated with the sections at 4 °C for 16 h. The sections were washed with 1 × PBS 3 times for 10 min and incubated with secondary antibody Alexa fluor 488 donkey anti-rabbit highly cross-adsorbed secondary antibody (1:400 dilution, A21206, Invitrogen, Carlsbad, CA) and Alexa fluor 555 donkey anti-goat cross-adsorbed secondary antibody (1:400 dilution, A21432, Invitrogen, Carlsbad, CA) for 1 h at 37 °C. The sections were observed by confocal microscope (TCS SP5, Leica, Wetzlar, Germany). Each brain has at least three sections and each section has two random fields of view.

### Western blot

The brain tissues in the peri-focal region were collected 14 days after tMCAO and added to the protein lysis solution (RIPA, Millipore, Billerica, MA). The tissue is subjected to 60 Hz frequency high-throughput tissue grinding for 45 s. The protein supernatants were collected by centrifugation at 12,000 ×*g* in 4 °C and stored at – 80 °C. Protein concentration was measured by the bicinchoninic acid assay (BCA, Thermo Fisher Scientific, Waltham, Massachusetts). The same amount of protein in each group was electrophoresed on a 10% separation gel (Epizyme, Shanghai, China). The protein is transferred from the separating gel to PVDF membrane, blocked by 10% protein-free protein blocking solution (Epizyme, Shanghai, China) and incubated with the primary antibodies against BDNF antibody (1:1000 dilution, sc-65514, Santa Cruz, Dallas, TX), bFGF antibody (1:1000 dilution, 05–118, Millipore, Billerica, MA), GDNF antibody (1:500 dilution, sc-13147, Santa Cruz, Dallas, TX) and β-actin (1:1000 dilution, 66,009, Proteintech, Rosemont, IL) at 4℃ over night. The transfer membrane was washed by 1 × TBST buffer (Meilunbio, Dalian, China), and incubated with horseradish peroxidase (HRP)-conjugated goat anti-mouse secondary antibody (1:5000 dilution, HA1006, Huabio, Hangzhou, China)for 1 h at room temperature. The expression of protein could be detected by highly sensitive ECL western blot substrate (Meilunbio, Dalian, China). Fluorescent density was measured with image J software.

### Dot blot

The PVDF membrane was activated by methanol for 5 min. We spotted 2 μL samples of PLGA, PLT@PLGA and RGD-PLT@PLGA onto a 0.45 μm PVDF membrane and dried it for 5 min at room temperature. The membrane was blocked with 5% skim milk for 30 min and washed with 1 × TBST three times. We incubated the membrane with primary antibody CD47 (1:1000 dilution, sc-12731, Santa Cruz, Dallas, TX) against specific region of extracellular immunoglobulin domain for two hours. The membrane was washed by 1 × TBST three times and incubated with horseradish peroxidase (HRP)-conjugated goat anti-rat secondary antibody (1:5000 dilution, HA1023, Huabio, Hangzhou, China)for 1 h at room temperature. The membrane was washed by 1 × TBST three times. The expression of protein could be detected by highly sensitive ECL western blot substrate (Meilunbio, Dalian, China) [52]. Fluorescent density was measured with image J software.

### Biodistribution of RGD-PLT@PLGA-FE in ischemic stroke mouse

To evaluate the biodistribution of RGD-PLT@PLGA-FE nanoparticles in ischemic stroke mice, the nanoparticles were prelabeled with DiD-dye that denoted as RGD-PLT@DiD/PLGA-FE. 24 h after tMCAO, RGD-PLT@DiD/PLGA-FE was injected via the tail vein. 24 h, 48 h and 72 h after injection, 30 μl of whole blood was collected via submandibular puncture and brain, lung, liver, spleen, and kidney were dissected, weighed, and homogenized in 1 × PBS (1 g tissue added with 1 ml 1 × PBS). Before measurements, the 30 μl whole blood was mixed well with 70 μL water. DiD fluorescence intensity in all samples was measured by a multiple-reader (Infinite M200, Tecan, Swiss) at an excitation/emission of 630/670 nm. PLGA encapsulated with FE and DiD (denoted as DiD/PLGA-FE) were served as the control. Fluorescence intensity per gram of tissue and relative signal per organ were calculated.

### Biocompatibility analysis of RGD-PLT@PLGA-FE in vivo

To assess the biocompatible property of RGD-PLT@PLGA-FE nanoparticles, healthy ICR mice received at the dose of 200 mg/kg of nanoparticles (30 mg/ml) (referred to PLT membrane protein) through the tail vein. At 2 days post injection, 600 μl whole blood was collected through the submandibular puncture and allowed to coagulate. After centrifugation at 3000 rpm for 5 min, serum was harvested for biochemistry markers measurement by Automatic biochemical analyzer (BX-3010, Sysmex Corporation, Kobe, Japan). Meanwhile, 50 μl of whole blood was collected into an anti-coagulation tube and then subjected to test blood routine by Hematology Analyzer (pocH-100iV, Sysmex Corporation, Kobe, Japan). After blood collection, the mice were sacrificed and the major organs including brain, lung, liver, spleen, and kidney were sliced to perform H&E staining for histological analysis.

### Statistical analysis

All calculated data were expressed as mean ± standard deviation (SD), and the statistical analysis was performed using one-way ANOVA for comparison of multiple groups. All the data were analyzed using Prism 9.0 (version 9.0.2). Statistical significance was considered when *p < 0.05, **p < 0.01, and ***p < 0.005.

## Supplementary Information

**Additional file 1:** **Figure S1.** The encapsulation efficiency and loading efficiency of FE. **Table S1.** Death rate of each group after tMCAO. **Figure S2.** The death and survival rate of mice after tMCAO in each group. **Figure S3.** Characterization of the insertion of RGD onto PLT membrane. **Figure S4.** The total fluorescence intensity in liver and spleen (RES). **Figure S5.** Cell proliferation and cytotoxicity assay. **Figure S6.** The protein compositions before and after the process of encapsulation. **Figure S7.** The stability of nanoparticles in solution. **Figure S8.** TTC staining assay.

## Data Availability

The datasets used and/or analyzed during the current study are available from the corresponding author on reasonable request.

## Abbreviations

FE: Fat Extract
PLTs: Platelets
RGD: Arg-Gly-Asp
PLGA: Poly(lactic-co-glycolic acid)
t-PA: Tissue plasminogen activator
ADSCs: Adipose derived stem cells
PTX: Paclitaxel
PCL: Polycaprolactone
iRGD: CRGD[K/R]GP[D/E]C
ICG: Indocyanine green
FL/PA: Fluorescence/photoacoustic
RvD2: Resolvin D2
TEM: Transmission electron microscopy
DLS: Dynamic light scattering
DiO: 3,3′-Dioctadecyloxa carbocyanine perchlorate
DiD: 1,1’-Dioctadecyl-3,3,3’,3’-Tetramethylindodicarbocyanine
RES: Reticuloendothelial system
mNSS: Modified neurological severity score
SIRPα: Signal regulatory protein α
RT: Room temperature
PVA: Polyvinyl alcohol
SDS-PAGE: Sodium dodecyl sulfate–polyacrylamide gel electrophoresis
PLT ghost: PLT vesicles
HRP: Horseradish peroxidase
FBS: Fetal bovine serum
SA: Scratch areas
tMCAO: Transient middle cerebral artery occlusion
ECA: External carotid artery
ICA: Internal carotid artery
MCA: Middle cerebral artery
EBST: Elevated body swing test
HPLC: High-performance liquid chromatography
