# Supplementary material for: Targeted delivery of fat extract by platelet membrane-cloaked nanocarriers for the treatment of ischemic stroke
Source: J Nanobiotechnology. 2022 May 31;20:249. doi: 10.1186/s12951-022-01461-2 (PMC9153102; doi:10.1186/s12951-022-01461-2)
Supplement: Supplementary file 1 — Additional file 1: Figure S1. The encapsulation efficiency and loading efficiency of FE. Table S1. Death rate of each group after tMCAO. Figure S2. The death and survival rate of mice after tMCAO in each group. Figure S3. Characterization of the insertion of RGD onto PLT membrane. Figure S4. The total fluorescence intensity in liver and spleen (RES). Figure S5. Cell proliferation and cytotoxicity assay. Figure S6. The protein compositions before and after the process of encapsulation. Figure S7. The stability of nanoparticles in solution. Figure S8. TTC staining assay. [file 12951_2022_1461_MOESM1_ESM.pdf]

Additional File 1

Targeted Delivery of Fat Extract by Platelet Membrane-Cloaked Nanocarriers for the Treatment of Ischemic Stroke

Cheng Wang[1#], Xuewei Yang[2#], Yixu Jiang[1], Lin Qi[1], Deli Zhuge[2], Tongtong Xu[1], Yiyan Guo[1], Mingwu Deng[3], Wenjie Zhang[3], Dongyan Tian[2], Qingqing Yin[2], Li Li[2], Zhijun Zhang[1], Yongting Wang[1], Guo-Yuan Yang[1], Yijie Chen[2]*, Yaohui Tang[1]*

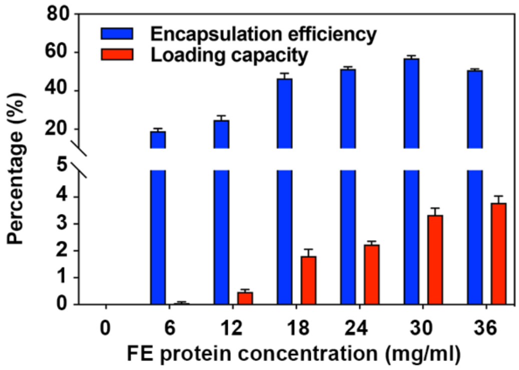

Supplementary Fig 1. The encapsulation efficiency and loading efficiency of FE.

The optimized FE loading efficiency and capacity were determined when the initial input of FE was up to 30 mg/ml (total protein concentration). Encapsulation efficiency (EE%) was calculated by (total protein input – free protein in supernatant) divided by the total protein added. Loading capacity (LC%) was calculated by (total protein added – free protein in the supernatant) divided by the total weight of PLGA plus entrapped protein. n=3/group. All data presented as mean ± SD.

Supplementary Table 1. Death rate of each group after tMCAO

Groups	10% Sucrose	FE only	PLGA only	PLGA-FE	PLT@PLGA	PLT@PLGA-FE	RGD-PLT@PLGA	RGD-PLT@PLGA-FE
Death rate (%)	33.33	22.22	22.22	22.22	33.33	21.43	23.08	26.67

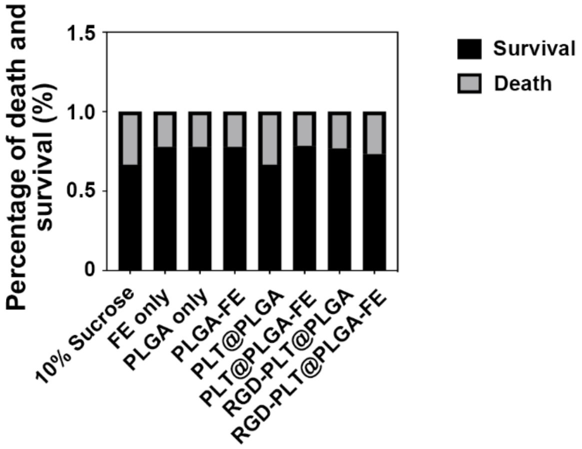

Supplementary Fig 2. The death and survival rate of mice after tMCAO in each group.

The death and survival rate in each group after tMCAO.

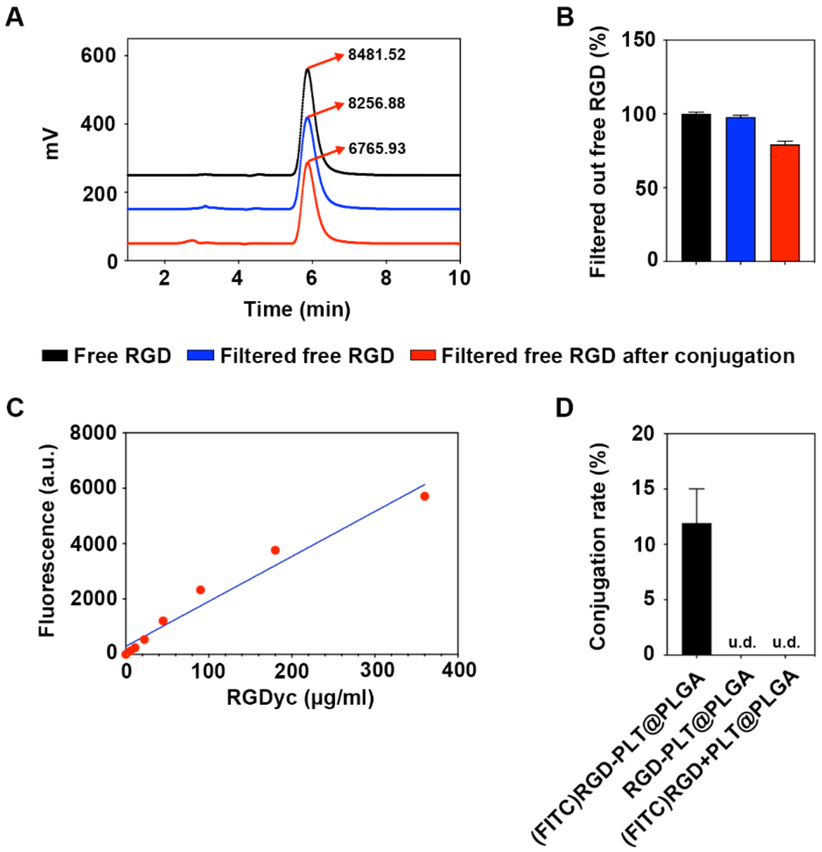

Supplementary Fig 3. Characterization of the insertion of RGD onto PLT membrane.

A. HPLC curves for free RGD (black line), filtered free RGD (blue line), and filtered free RGD after conjugation (red line). n = 3/group. B. Percentages of filtered out free RGD in three groups. n

= 3/group. C. The standard curve generated from (FITC)RGD at various concentrations measured by a microplate reader at an excitation/emission of 488/525 nm. n = 3/group. D. Conjugation rate of (FITC)RGD to (FITC)RGD-PLT@PLGA. RGD-PLT@PLGA and (FITC)RGD+PLT@PLGA were employed as controls. n = 3/group. Data presented as mean ± SD.

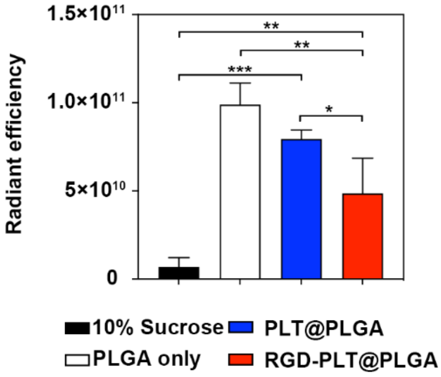

Supplementary Fig 4. The total fluorescence intensity in liver and spleen (RES).

Quantitative analysis of the average DiD fluorescence signal intensities in the liver and spleen that treated with 10% sucrose, PLGA, PLT@PLGA and RGD-PLT@PLGA. n=3/group. Data presented as mean ± SD. *$p < 0.05$, **$p < 0.01$, ***$p < 0.001$.

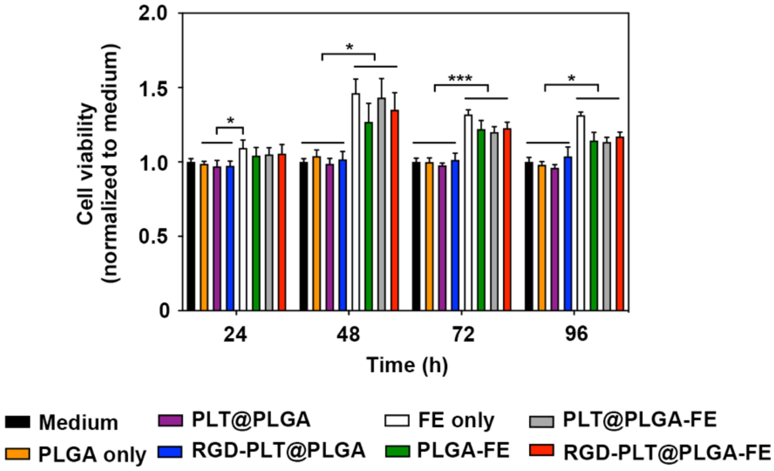

Supplementary Fig 5. Cell proliferation and cytotoxicity assay.

Normalized of cell viability that treated with medium, FE, PLGA, PLGA-FE, PLT@PLGA,

PLT@PLGA-FE, RGD-PLT@PLGA, RGD-PLT@PLGA-FE over time (24 h, 48 h, 72 h and 96 h). n=3/group. Data presented as mean ± SD. *p< 0.05, ***p< 0.001

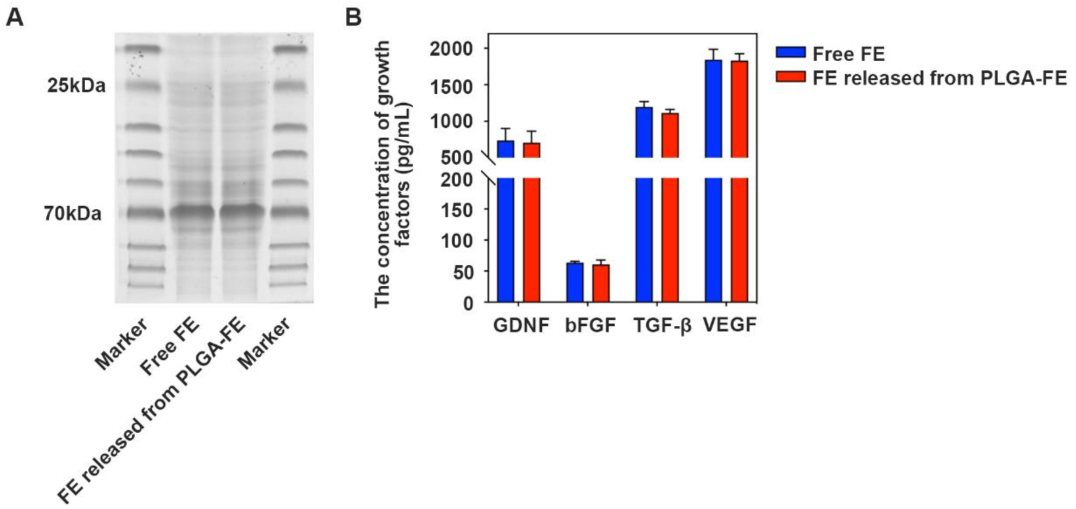

Supplementary Fig 6. The protein compositions before and after the process of encapsulation.

A. Protein compositions of FE before and after the process of encapsulation by Coomassie staining.

B. Quantitative analysis of the concentration of growth factors including GDNF, bFGF, TGF-β and VEGF in FE either before or after encapsulation by ELISA. n=5/group. Data presented as mean ± SD.

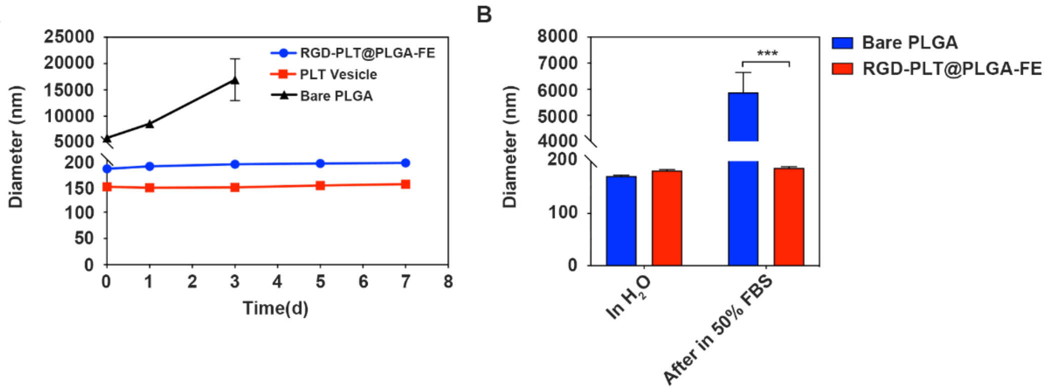

Supplementary Fig 7. The stability of nanoparticles in solution.

A. The diameter of bare PLGA, PLT Vesicle and RGD-PLT@PLGA-FE over 0d, 1d, 3d, 5d, 7d.

n=3/group. B. The diameter of bare PLGA and RGD-PLT@PLGA-FE in water and 50% FBS. n=3/group. Data presented as mean ± SD. ***$p < 0.001$.

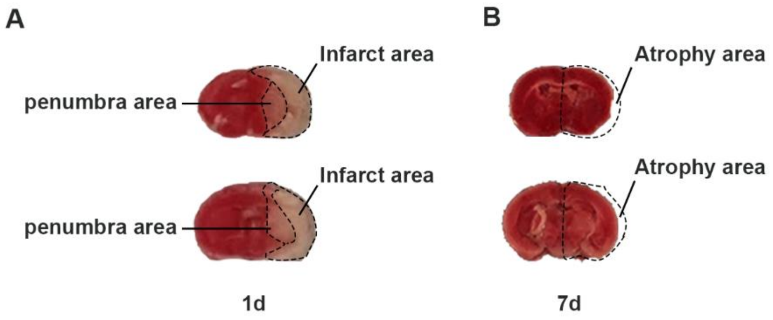

Supplementary Fig 8. TTC staining assay.

A. The TTC staining for ischemic stroke on day 1. B. The TTC staining for ischemic stroke on day 7.
